# Sensitive photodetection below silicon bandgap using quinoid-capped organic semiconductors

**DOI:** 10.1126/sciadv.adf6152

**Published:** 2023-03-29

**Authors:** Tengfei Li, Gangjian Hu, Liting Tao, Jizhong Jiang, Jingming Xin, Yawen Li, Wei Ma, Liang Shen, Yanjun Fang, Yuze Lin

**Affiliations:** ^1^ Beijing National Laboratory for Molecular Sciences, CAS Key Laboratory of Organic Solids, Institute of Chemistry, Chinese Academy of Sciences, Beijing, China.; ^2^ State Key Laboratory of Integrated Optoelectronics, College of Electronic Science and Engineering, International Center of Future Science, Jilin University, Changchun, China.; ^3^ State Key Laboratory of Silicon Materials, School of Materials Science and Engineering, Zhejiang University, Hangzhou, China.; ^4^ State Key Laboratory for Mechanical Behavior of Materials, Xi’an Jiaotong University, Xi’an, China.; ^5^ University of Chinese Academy of Sciences, Beijing, China.

## Abstract

High-sensitivity organic photodetectors (OPDs) with strong near-infrared (NIR) photoresponse have attracted enormous attention due to potential applications in emerging technologies. However, few organic semiconductors have been reported with photoelectric response beyond ~1.1 μm, the detection limit of silicon detectors. Here, we extend the absorption of organic small-molecule semiconductors to below silicon bandgap, and even to 0.77 eV, through introducing the newly designed quinoid-terminals with high Mulliken-electronegativity (5.62 eV). The fabricated photodiode-type NIR OPDs exhibit detectivity (D^*^) over 10^12^ Jones in 0.41 to 1.2 μm under zero bias with a maximum of 2.9 × 10^12^ Jones at 1.02 μm, which is the highest D^*^ for reported OPDs in photovoltaic-mode with response spectra beyond 1.1 μm. The high D^*^ in 0.9 to 1.2 μm is comparable to those of commercial InGaAs photodetectors, despite the detection limit of our OPDs is shorter than InGaAs (~1.7 μm). A spectrometer prototype with a wide measurable region (0.4 to 1.25 μm) and NIR imaging under 1.2-μm illumination are demonstrated successfully in OPDs.

## Teaser

Organic photodetectors show detectivities >10^12^ Jones in 0.41-1.2 μm, better than or comparable to the commercial detectors.

## INTRODUCTION

Sensitive near-infrared (NIR) light detection is central to modern science and technology, such as night vision, health monitoring, biological/medical imaging, optical communications, three-dimensional object recognition, and artificial intelligence (*1*–*4*). Current NIR photodetectors (PDs) mainly use photodiodes based on crystalline inorganic semiconductors (*2*, *5*), such as silicon (Si) (*6*), germanium (Ge) (*7*), and III to V compounds (*8*). Solution-processed NIR-sensitive semiconductors, including organic materials, quantum dots, and hybrid perovskite materials, have emerged as candidates for next-generation light sensing, which combine low cost, ease of processing, facile integration with complementary metal oxide semiconductors, tailorable optoelectronic properties, and compatibility with flexible substrates (*1*–*3*, *9*, *10*). Different from the other solution processed materials like metal halide perovskites and quantum dots, only organic semiconductors have no heavy metal toxicity. In past years, NIR organic photodetectors (OPDs) have received broad attention and shown comparable sensitivity to those of inorganic/hybrid PDs at the wavelength of less than 1.0 to 1.1 μm (*4*, *11*), which is the detection limit prevented by electronic bandgap of widely used single crystal Si in modern electronics. However, very limited successful strategies have been reported to extend the response range of NIR OPDs to over 1.1 μm along with high sensitivity (*12*), which is necessary to be complementary to silicon electronics industry.

**Fig. 1.**
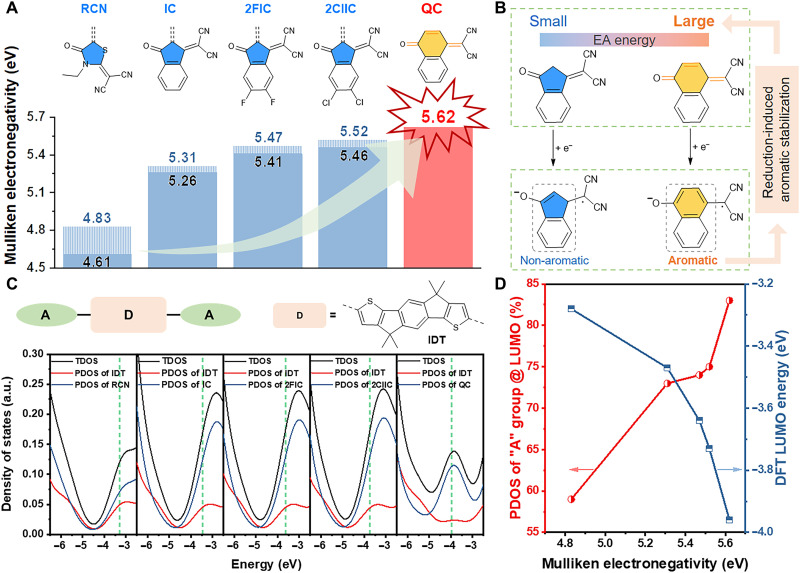
High Mulliken electronegativity of QC. (**A**) The chemical structures and Mulliken electronegativity of QC and other widely used “A” groups (the black values are for widely used “A” groups, and the blue values are for their vinyl-contained derivatives). (**B**) The one-electron reduction of IC group with small vertical EA energy and reduction-induced aromatic stabilization of QC group with large vertical EA energy. (**C**) Total density of states (TDOS) and partial DOS (PDOS) of IDT-based A-D-A molecules with QC and other widely used “A” groups calculated by Gaussian 16 program at B3LYP/6-31G(d,p) level (green dashed line: LUMO). (**D**) The relationship between the PDOS contribution of “A” group to LUMO (red symbol and line) and the calculated LUMO energy (blue symbol and line) of IDT-based A-D-A molecules with QC and other widely used “A” groups and the Mulliken electronegativity of corresponding “A” groups. a.u., arbitrary units.

One of the greatest challenges for high-sensitivity NIR OPDs is the design and synthesis of high-performance organic photoactive materials with ultra-narrow bandgap (*13*). Initially, the research was mainly focused on conjugated polymers through extending the conjugation length, minimizing the bond-length alternation, and/or enhancing the donor-acceptor charge transfer effect (*14*, *15*). Although the bandgap as low as ~0.5 eV can be achieved (*16*), the intrinsic drawbacks of the large structure disorder and poor batch-to-batch replicability of the polymer quality (e.g., molecular weight and polymer dispersity index) restrict the performance and commercialization of OPDs based on NIR-absorbing polymers (*11*). In contrast, small-molecule semiconductors with well-defined structures are more promising to fabricate commercial high-sensitivity OPDs. Nowadays, the acceptor-donor-acceptor (A-D-A) concept has been proved to be one of the most fruitful molecular design strategies to construct small-molecule semiconductors with tuned bandgaps (*17*), where their molecular orbitals are formed through the hybridization of original noninteracting orbitals of the electron-donating “D” and electron-withdrawing “A” segments (*18*). Cyano (CN)–contained units were the most widely used “A” segments, including 2-(3-ethyl-4-oxothiazolidin-2-ylidene)malononitrile, 3-(1,1-dicyanomethylene)-1-indanone (IC), 3-(1,1-dicyanomethylene)-5,6-difluoro-1-indanone (2FIC), and 3-(1,1-dicyanomethylene)-5,6-dichloro-1-indanone (2ClIC) (Fig. 1A) (*19*–*23*). However, these existing “A” groups are hardly capable to extend the absorption of organic semiconductors to over 1.1 μm, due to the limited strength of their electron withdrawing properties (*24*, *25*). Therefore, designing ultrastrong electron-withdrawing “A” group becomes the key point to constructing ultra-narrow bandgap small-molecule semiconductors.

Here, we develop an efficient molecular design strategy to construct ultra-narrow bandgap small-molecule semiconductors by introducing a cyano-contained quinoid capping group, which shows much stronger electron-withdrawing property than those of existing “A” groups. The obtained semiconductors show ultra-narrow optical bandgaps as low as 0.77 eV, with a large absorption onset of ~1.7 μm. Photodiode-type NIR OPDs based on the designed NIR-sensitive molecules show the detectivity as high as 2.9 × 10^12^ Jones at 1.02 μm under zero bias at room temperature, which is the highest value reported for NIR OPDs operating in photovoltaic mode with response spectra below Si bandgap so far (*26*–*28*). The high detectivity we obtained is one order higher than commercial Ge detector and even on a par with those of commercial InGaAs PDs in the wavelength range of 0.9 to 1.2 μm (*29*–*32*). Furthermore, spectrometer with a wide measurable region of 0.4 to 1.25 μm and NIR imaging under 1.2-μm wavelength illumination are both achieved by using solution-processed NIR OPDs made of molecular semiconductors we developed here, which is rarely achieved by reported OPDs before.

## RESULTS AND DISCUSSION

### Design strategy and properties of NIR molecular semiconductors

Quinones with two carbonyl groups in an unsaturated six-membered ring structure generally have an electron-poor π-orbital system and exhibit deep lowest unoccupied molecular orbital (LUMO) energy level (*33*, *34*). Naphthalene-1,4-dione, as a stabilized quinoid and easily available material, has been widely used in medicine, dye, and other industries (*35*). Taking the strong electron-withdrawing property of −CN group into account, the combination of quinoid feature and −CN unit is promising to be an effective strategy to construct strong electron-deficient “A” groups, which can further acquire A-D-A small-molecule semiconductors with ultra-narrow bandgap.

The Mulliken electronegativity is generally used to probe the electron-withdrawing ability of a molecule or segment, which is defined as half of the sum of the vertical electron affinity (EA) energy and the vertical ionization potential (*36*). According to the molecular modeling by density functional theory (DFT), 2-[4-oxonaphthalen-1(4*H*)-ylidene]malononitrile (QC) (Fig. 1A) derived from naphthalene-1,4-dione and malononitrile has much higher Mulliken electronegativity of 5.62 eV (Fig. 1A), relative to naphthalene-1,4-dione (5.21 eV) and malononitrile (5.05 eV). Moreover, the Mulliken electronegativity of QC is also higher than those of the forementioned widely used “A” groups (4.61 to 5.46 eV; Fig. 1A) and their vinyl-contained derivatives (4.83 to 5.52 eV; Fig. 1A). The high Mulliken electronegativity nature of QC group is mainly related with its larger vertical EA energy (2.29 eV) relative to the forementioned widely used “A” groups (1.10 to 2.10 eV), which originates in the reduction-induced aromatic stabilization. Upon one-electron reduction, QC can generate the aromatic naphthalenoid substructure with radical anion character, while the one-electron reduction products of the forementioned widely used “A” groups are less stable (Fig. 1B) (*37*). To the best of our knowledge, the Mulliken electronegativity of the newly designed QC unit (5.62 eV) is the highest value for building blocks within organic semiconductors, confirming that the QC unit is expected to be an excellent “A” group that has ultrastrong electron-withdrawing ability and can construct NIR-absorbing small-molecule semiconductors.

To check the capability of QC in narrowing bandgap, DFT calculation was carried out on several indacenodithiophene (IDT)–based A-D-A molecules with varied “A” groups, where the most widely used “A” groups were investigated as references. First, the contribution to the LUMO of the “D” and “A” segments of the molecular backbone was quantitatively evaluated through the calculation of total density of states (TDOS) and partial DOS (PDOS) (Fig. 1C and table S1). Because of the ultrastrong electron-deficient ability of the QC group, the contribution of terminal units to LUMO in QC-based molecule is as large as 83% and much higher than those (59 to 75%) in reference molecules (table S1). Relative to the reference molecules, the QC-based molecule exhibits an obviously deeper LUMO energy level (∆LUMO of 0.23 to 0.68 eV) as well as a higher lying highest occupied molecular orbital (HOMO) (fig. S1). Larger Mulliken electronegativity of terminal units leads to more DOS contribution of “A” group to LUMO and deeper LUMO energy level (Fig. 1D), which is beneficial to acquire a narrower bandgap for A-D-A molecules. As a result, The QC-based molecule shows a notably smaller HOMO-LUMO gap than those of other calculated molecules based on the other “A” groups. Moreover, the similar trend in energy levels is also observed in indacenodithieno[3,2-*b*]thiophene–based A-D-A molecules (fig. S2), which indicates that much narrower bandgaps could be acquired in QC-based organic semiconductors commonly, relative to the control molecules with traditional “A” groups.

**Fig. 2.**
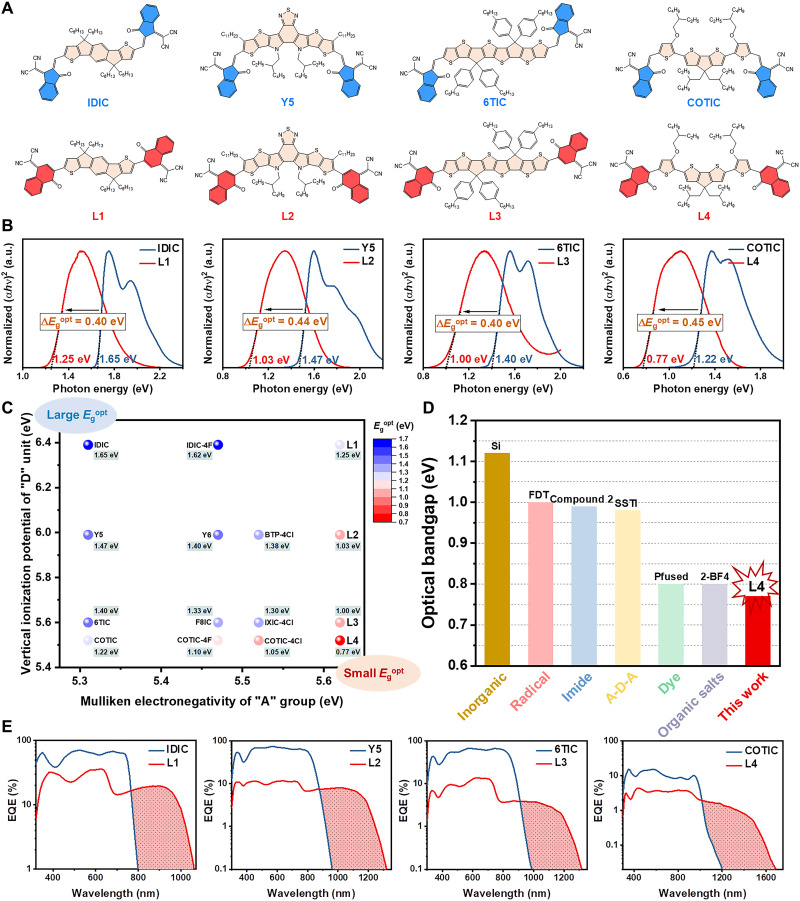
Narrow bandgap of QC-based molecules. (**A**) The chemical structures of L1 to L4 and their IC-based analogs. (**B**) The absorption spectra of CHCl_3_-cast thin films with the corresponding optical bandgaps and the ∆*E*_g_^opt^ values between L1 to L4 and their IC-based analogs. (**C**) The summary of optical bandgap values (obtained from the absorption spectra of thin film) of A-D-A small-molecule semiconductors which consist of various “D” units (IDT, BTP, 3TT, and COT with different vertical ionization potential of 6.39, 5.99, 5.60, and 5.52 eV) and “A” groups (QC, IC, 2FIC, and 2ClIC). (**D**) The summary of reported organic photovoltaic small-molecule semiconductors with small optical bandgaps. Single crystal Si is used as the reference, as well as some molecular semiconductors with the smallest optical bandgap among their corresponding type of compounds. (**E**) EQE spectra comparison of NIR OPDs based on L1 to L4 and their IC-based analogs with device structure of ITO/PEDOT:PSS/active layer/DPO/Al under zero bias.

Coincidently, the QC-based coupling compound is the analog of the condensation compound based on IC group. To illustrate the capability of QC unit in narrowing the optical bandgap intuitively, four A-D-A compounds (L1, L2, L3, and L4) composed of QC end groups with various electron-rich cores {IDT, dithienothiophen[3,2-*b*]pyrrolobenzothiadiazole (BTP), terthieno[3,2-*b*]thiophene (3TT), and 2,6-bis(3-alkoxythiophen-2-yl)-cyclopentadithiophene (COT); fig. S3A} were synthesized and compared with their IC-based analogs [IDIC (*38*), Y5 (*39*), 6TIC (*40*), and COTIC] (Fig. 2A). The detailed synthetic procedures are shown in fig. S3A. Two synthetic routes for QC-based semiconductors are used here: (i) Stille coupling reaction between the distannyl derivative of electron-rich core and 2-bromonaphthalene-1,4-dione and then condensation reaction using malononitrile (synthetic route for L1); (ii) condensation reaction between 2-bromonaphthalene-1,4-dione and malononitrile followed by Stille coupling reaction using the distannyl derivative of electron-rich core (synthetic routes for L2, L3, and L4).

As shown in fig. S3B, all the absorption spectra of QC-based semiconductors red shift a lot relative to those of their IC-based analogs. In dilute CHCl_3_ solution (~10^−6^ M), the long-wavelength absorption peaks (λ_max_s) of L1 to L4 are located at 812, 878, 958, and 1152 nm, respectively, while their IC-based analogs show blue-shifted absorption region with ∆λ_max_s of 144, 158, 188, and 302 nm, respectively. The thin films of L1 to L4 exhibit red-shift absorption spectra along with λ_max_ values of 832, 969, 988, and 1298 nm, respectively, relative to those of their IC-based analogs which are 713, 780, 800, and 914 nm, respectively (fig. S3B). It is worth noting that the neat films of L2 to L4 show absorption onsets of ~1.3, ~1.3, and ~1.7 μm, respectively, which are all beyond the photoresponse limit of single crystal Si (fig. S3B). Moreover, all the films of QC- and IC-based materials exhibit varying degrees of redshift on the absorption spectra, relative to those of their dilute solutions, which is related to the molecular *J*-aggregation in thin films (*41*). In Kasha’s exciton model, the redshift originates from the negative Coulomb coupling between two molecules, as determined by the slip-stacked packing arrangement (*41*). Grazing-incidence wide-angle x-ray scattering (GIWAXS) (*42*) of the thin film was carried out taking L2 and Y5 as the representative to study the molecular packing in aggregate states of QC- and IC-based materials (fig. S4). Both L2 and Y5 exhibit dominant “face-on” orientation with sharp (010) π-π stacking peaks at *q*_z_ ≈ 1.69 Å^−1^ (*d* = 3.72 Å) for L2 and *q*_z_ ≈ 1.74 Å^−1^ (*d* = 3.61 Å) for Y5 (fig. S4). The similar molecular skeleton and *d* spacing of π-π stacking lead to the almost same redshift degree of 0.13 eV of the neat film relative to their dilute solution (fig. S3B) (*43*).

As depicted in Fig. 2B, L1 to L4 have optical bandgaps (*E*_g_^opt^s) of 1.25, 1.03, 1.00, and 0.77 eV, respectively, which are obviously smaller than those of their IC-based analogs (1.65, 1.47, 1.40, and 1.22 eV). According to cyclic voltammetry (CV) measurement and DFT calculation (fig. S5), the smaller *E*_g_^opt^ of QC-based semiconductors can be attributed to notably deeper LUMO and slightly shallower HOMO energy levels than those of their IC-based analogs. Meanwhile, L1 to L4 all exhibit quasi-reversible reduction waves in CV measurement, while their IC-based analogs show irreversible ones. The better electrochemical reduction stability of L1 to L4 may be attributed to the reduction-induced aromatic stabilization of QC group (Fig. 2B). Moreover, all the four compounds exhibit high decomposition temperature at 5% weight loss of over 300°C, indicating that L1 to L4 all have excellent thermal stabilities (fig. S6A). The photo-oxidation stabilities were also probed through the absorption decay of the corresponding film upon illumination with AM 1.5G (100 mW cm^−2^) in the ambient air (fig. S6, B to F). After continuous illumination for 20 hours, the absorption peak intensities of the four films decay to 86 to 92% of the initial values, implying good photo-oxidation stability.

To reveal the effect of electronegativity of “A” group on the *E*_g_^opt^ of A-D-A semiconductors, the *E*_g_^opt^ values of a series of materials with various electron-rich cores (IDT, BTP, 3TT, and COT) and “A” terminals (IC, 2FIC, 2ClIC, and QC) were summarized (Fig. 2C and table S2). As shown in Fig. 2C, with the enhanced Mulliken electronegativity of “A” terminals, the *E*_g_^opt^s of the A-D-A compounds gradually reduce, given that the electron-rich core is the same. Moreover, the bandgaps of QC-based compounds reduce by similar ∆*E*_g_^opt^ values, relative to those compounds based on IC (∆*E*_g_^opt^ of 0.4 to 0.45 eV), 2FIC (∆*E*_g_^opt^ of 0.33 to 0.37 eV), or 2ClIC (∆*E*_g_^opt^ of 0.28 to 0.35 eV), independent of electron-rich cores (table S2). The results above confirm that the highly electronegative QC end group can quantitatively narrow bandgaps of A-D-A molecules through replacing traditional end-capped groups to some extent. To the best of our knowledge, the 0.77 eV is the smallest *E*_g_^opt^ value reported for organic photovoltaic small-molecule semiconductors, including A-D-A molecules, dyes, and other kinds of molecules (Fig. 2D and table S3). Such small bandgaps along with good thermal and chemical stabilities endow these QC-based semiconductors with the enormous potential in the application of NIR PDs, such as optical imaging and spectrometer.

### Device performance of NIR OPDs

To demonstrate potential application in NIR OPDs of QC-based ultra-narrow bandgap molecules with low energy levels, photodiode-type devices were fabricated by blending polymer donors (fig. S7A) with relatively high energy levels to form bulk heterojunction for efficient exciton dissociation (*44*, *45*). The conventional structure of indium tin oxide (ITO) /poly(3,4-ethylenedioxythiophene):poly-(styrenesulfonate) (PEDOT:PSS) /active layer/{[2-(1,10-phenanthrolin-3-yl)naphth-6-yl]diphenylphosphine oxide} (DPO) (*46*) /Al was first used. The external quantum efficiency (EQE) spectra of the OPDs based on L1 to L4 and their IC-based analogs are summarized in Fig. 2E. Relative to the spectral response ranges of the OPD devices based on their IC-based analogs, L1- to L4-based OPDs exhibit notably red-shifted response range, which is in accordance with the change in their absorption spectra range. Responsivity (*R*) is defined as the ratio of the photocurrent to the incident light intensity and can be calculated as

$$R=\frac{\mathrm{EQE}}{100\%}\times \frac{\lambda }{1240}$$ (1)

where λ is the wavelength of incident light in nanometer. Among these OPDs, PDPPDTP (*47*):L4–based devices show the broadest response spectra approaching 1.7 μm, and PTB7-Th (*48*):L2–based devices exhibit the highest sensitivity beyond the wavelength of 1.1 μm, which is the response limitation of the most-widely used commercial Si-based PDs (fig. S7B). Therefore, the OPD devices based on PTB7-Th:L2 were used to further study the performance and practical application of photodetection.

For an OPD device, the specific detectivity (D^*^) is the key figure of merit to quantify the ability to detect faint light signals, which can be described by the following equation (*49*)

$$D^{*}=\frac{\sqrt{A}}{\mathrm{NEP}}=\frac{R\sqrt{AB}}{i_{n}}$$ (2)

where *A* is the device area (0.04 cm^2^ in this work), NEP is the noise equivalent power, *B* is the electrical measurement bandwidth, and *i*_n_ is the noise current. To acquire a high D^*^ value, both high photoresponse and low *i*_n_ value are required. The *i*_n_ for a photodiode-type OPD can be given as following equations (*50*)

$$i_{n}=\sqrt{i_{\mathrm{shot}}^{2}+i_{\mathrm{thermal}}^{2}+i_{1/f}^{2}}$$ (3)

$$i_{\mathrm{shot}}=\sqrt{2eI_{d}B}$$ (4)

$$i_{\mathrm{thermal}}=\sqrt{\frac{4k_{B}TB}{R_{\mathrm{sh}}}}$$ (5)

where *i*_shot_ is the shot noise, *i*_thermal_ is the thermal noise, *i*_1/f_ is the 1/f noise, *e* is the elementary charge, *I*_d_ is the dark current, *k*_B_ is the Boltzmann constant, *T* is the absolute temperature, and *R*_sh_ is the shunt resistance of the device.

To acquire the D^*^ value, EQE and responsivity of the NIR OPDs were first probed. PTB7-Th:L2–based conventional devices exhibit broad spectra photoresponse with EQE values over 6% from 0.32 to 1.13 μm and the maximum *R* value is 0.067 A W^−1^ at the wavelength of 1.02 μm under zero bias (Fig. 2E and fig. S7B). The detailed performance parameter of L2-based NIR OPDs can be seen in Table 1. It is worth noting that plenty of reported results on OPDs in literatures suppose that the noise power spectrum is “white” and calculated from the root mean square of *i*_shot_ and *i*_thermal_ (even only using *i*_shot_ as the total noise current), generally leading to overestimated D^*^ values (*1*, *51*). For precisely evaluating the detectivity of an OPD device, the frequency dependence of the noise current is required to be fully considered (*49*). As shown in fig. S8A, the noise current is frequency independent in the test frequency range and evaluated as an average value of 3.5 × 10^−14^ A Hz^−1/2^ under zero bias. The D^*^ related with *R* and *i*_n_ can be calculated according to the Eq. 2, and the conventional devices based on PTB7-Th:L2 exhibit D^*^ values above 10^11^ Jones in the range of 0.3 to 1.2 μm with the highest value of 3.8 × 10^11^ Jones at 1.02 μm under zero bias (fig. S8A).

**Table 1.** Detailed performance parameters of L2-based NIR OPDs. The active area of the device is 0.04 cm^2^. The *R* and *i*_n_ are measured in the ambient air at room temperature.

Device structure	Bias* (V)	*R*† (A W^−1^)	*i*_n_‡ (A Hz^−1/2^)	Detectivity§ (Jones)
ITO/PEDOT:PSS/PTB7-Th:L2/DPO/Al	0	0.067	3.5 × 10^−14^	3.8 × 10^11^ at 1020 nm
ITO/ZnO/PTB7-Th:L2/MoO_3_/Ag	0	0.073	7.0 × 10^−15^	2.1 × 10^12^ at 1020 nm
ITO/ZnO/PTB7-Th:L2/MoO_3_/Ag	−1	0.090	1.2 × 10^−13^	1.5 × 10^11^ at 980 nm
ITO/ZnO/PTB7-Th:L2/MoO_3_/Ag	−3	0.123	2.6 × 10^−12^	9.5 × 10^9^ at 980 nm
ITO/ZnO/PTB7-Th:L2:Y6/MoO_3_/Ag	0	0.084	5.7 × 10^−15^	2.9 × 10^12^ at 1020 nm

*The bias voltage applied on the device.

†The maximum responsivity in the NIR region.

‡The real-measured noise current.

§The maximum D* in the NIR region with the corresponding wavelength.

**Fig. 3.**
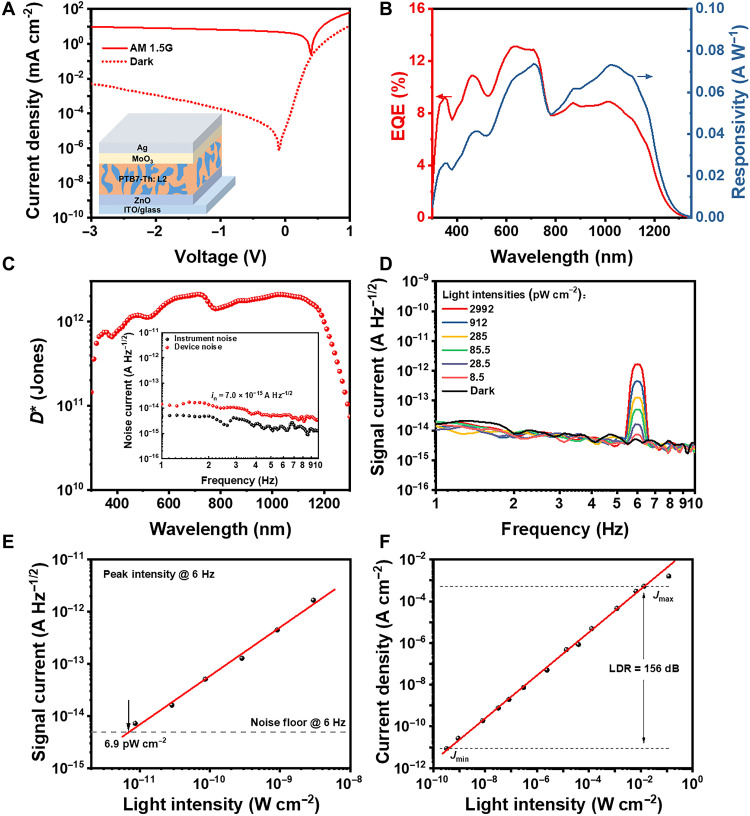
Performance of NIR OPDs. (**A**) Characteristic *J-V* curves under AM 1.5G irradiation (100 mW cm^−2^) and in the dark (inset: schematic diagram of device structure). (**B**) EQE spectrum and responsivity under zero bias. (**C**) Specific detectivity under zero bias (inset: the measured device noise current under zero bias). (**D**) The signal current under zero bias under 1-μm light illumination modulated at 6 Hz with various light intensities. (**E**) The peak signal intensity at 6 Hz obtained from (D) as a function of light intensities. The solid line is a linear fitting to the data and the dashed line is the device noise level in the dark. (**F**) The linear dynamic range (LDR) measurement under the illumination at 1 μm of various light intensities under zero bias. The solid line is a linear fitting to the data.

According to the *J*-*V* characteristics in the dark (fig. S7C), *R*_sh_ can be deduced as 3.2 × 10^7^ ohm from the differential resistance (fig. S8B). Consequently, *i*_thermal_ of 2.3 × 10^−14^ A Hz^−1/2^ can be calculated from Eq. 5, which implies that the *i*_n_ value of our conventional OPD device is dominated by *i*_thermal_ under zero bias. To acquire a higher detectivity, the inverted device structure of ITO/ZnO/PTB7-Th:L2/MoO_3_/Ag (Fig. 3A) was applied, because the efficient charge-blocking ability of ZnO and MoO_3_ could effectively suppress the noise current, especially white noise (*52*).

Relative to conventional devices, the inverted OPD devices based on PTB7-Th:L2 exhibit similar spectra photoresponse with a maximum *R* value in the NIR region of 0.073 A W^−1^ at the wavelength of 1.02 μm under zero bias (Fig. 3B). When it comes to the noise current, the inverted devices exhibit almost frequency independent in the test frequency range and evaluated as an average value of 7.0 × 10^−15^ A Hz^−1/2^ under zero bias (Fig. 3C), which is only one fifth of the measured noise of conventional devices (3.5 × 10^−14^ A Hz^−1/2^). The deduced *R*_sh_ of 3.8 × 10^8^ ohm leads to *i*_thermal_ value of 6.6 × 10^−15^ A Hz^−1/2^, which indicates that the *i*_n_ value of inverted device is also dominated by *i*_thermal_ under zero bias (fig. S9). Thanks to the lower noise current relative to the conventional OPD, the inverted OPD devices based on PTB7-Th:L2 exhibit D^*^ values above 10^12^ Jones in the range of 0.44 to 1.2 μm with the highest value of 2.1 × 10^12^ Jones at 1.02 μm under zero bias (Fig. 3C). The D^*^ value of 2.1 × 10^12^ Jones is the highest detectivity reported for NIR OPDs operating in photovoltaic mode with photoresponse beyond 1.1 μm (below Si bandgap; table S4) (*26*–*28*).

According to the Eq. 2, the NEP value of our inverted NIR OPDs based on PTB7-Th:L2 is calculated to be 1.0 × 10^−13^ W Hz^−1/2^ at the wavelength of 1 μm (D^*^ = 2.0 × 10^12^ Jones), implying that the inverted OPDs based on PTB7-Th:L2 with a working area of 0.04 cm^2^ can detect the NIR light (1 μm) intensity as low as 2.5 pW cm^−2^. To verify whether the calculated NEP agrees well with the true detection limit of the NIR OPDs based on PTB7-Th:L2, the total currents under zero bias under 1-μm-wavelength illumination with different light intensity were recorded in the same way as the measurement of noise current (*53*). As depicted in Fig. 3D, a peak at the frequency of 6 Hz appears under the illumination and decreases gradually with reducing the light intensity. Figure 3E illustrates the irradiance-dependent signal intensity at 6 Hz, and the signal peak intensity decreases linearly with the light intensity. The device keeps the linear response down to 8.5 pW cm^−2^, which is the lowest light intensity of 1 μm light source we can obtain and accurately calibrate. The corresponding linear-extrapolated value is 6.9 pW cm^−2^ for inverted OPD devices that is close to the calculated value of 2.5 pW cm^−2^ (Fig. 3E).

As an important figure of merit for practical applications, the linear dynamic range (LDR), defined as the incident optical power range where the response of PDs is linear, can be calculated from the equation below (*54*)

$$\mathrm{LDR}=20\times \log\frac{J_{\max}}{J_{\min}}$$ (6)

where *J*_max_ and *J*_min_ are the current density values of the OPD device under the highest and the lowest light intensities in a particular range. As clearly shown in Fig. 3F, with the enhancement of light intensity from 0.32 nW cm^−2^ to 13 mW cm^−2^, the photocurrent of inverted devices based on PTB7-Th:L2 increases linearly from 8.6 pA cm^−2^ to 0.53 mA cm^−2^, leading to the LDR value of 156 dB under 1-μm-wavelength illumination under zero bias. Meanwhile, The LDR for conventional OPD devices is also acquired as 171 dB (fig. S8C). The LDR values of our PTB7-Th:L2–based OPD devices are a little lower than those of the commercial NIR PD based on Ge (~200 dB for Judson J16) (*29*) and Si (~220 dB for Hamamatsu S1787) (*55*) but higher than that of the reported InGaAs PD (132 dB) (*56*), calculated by the same method. The large LDR, along with high D^*^ in a wide spectra region, indicates that the NIR OPD based on PTB7-Th:L2 can be capable for practical applications, such as spectrometer and NIR imaging.

### Performance optimization of NIR OPDs

Energy gap law specifies that a faster nonradiative deactivation pathway in semiconductors with narrower bandgap drastically reduces the emission intensity (*57*), and narrower bandgap generally results in an increased nonradiative charge transfer state decay rate that also obeys an energy gap law, further leading to a less charge generation in longer wavelength systems (*58*). Consequently, OPDs based on NIR-absorbing materials generally exhibit low photoresponse, resulting in lower D^*^ values, compared with shorter wavelength OPDs. As an example, the inverted OPD devices based on PDPPDTP: L4 with broad response spectra, from 0.3 to 1.6 μm, show low EQE values of <2% in the NIR region, along with high measured noise current of 1.0 × 10^−12^ A Hz^−1/2^, leading to D^*^ values only ~10^9^ Jones under zero bias (fig. S10). Applying reverse bias (here −1 and − 3 V applied) is widely used to improve the charge-carrier extraction efficiency in OPDs, which is expected to enhance the EQE. Relative to those obtained under zero bias, both EQE intensities and *R* values of inverted OPD devices based on PTB7-Th:L2 obviously increase in the whole photoresponse region, reaching up to maximum values of 15.6% and 0.123 A W^−1^ at 980 nm in the NIR region under −3 V (fig. S11, A and B). The EQE values of much less than the unit achieved in NIR OPDs here should be at least partially attributed to the energy gap law.

However, under the reverse bias of −1 and −3 V, the *i*_n_ values of inverted OPD devices exhibit obvious frequency dependence in the whole test frequency range. The measured *i*_n_ values are obtained as 1.2 × 10^−13^ A Hz^−1/2^ (−1 V) and 2.6 × 10^−12^ A Hz^−1/2^ (−3 V) at 210 Hz (the test frequency of EQE) (fig. S11C), which are notably higher than the *i*_n_ under zero bias (7.0 × 10^−15^ A Hz^−1/2^). Consequently, the maximum D^*^ values in NIR region are calculated as 1.5 × 10^11^ Jones at 980 nm under −1 V and 9.5 × 10^9^ Jones at 980 nm under −3 V, respectively (fig. S11D). Despite the enhancement in EQE and responsivity under reverse bias, the concomitant improved *i*_n_ values lead to approximately one to two orders of magnitude reduction of detectivity for inverted OPDs, relative to the OPD operating at photovoltaic mode.

**Fig. 4.**
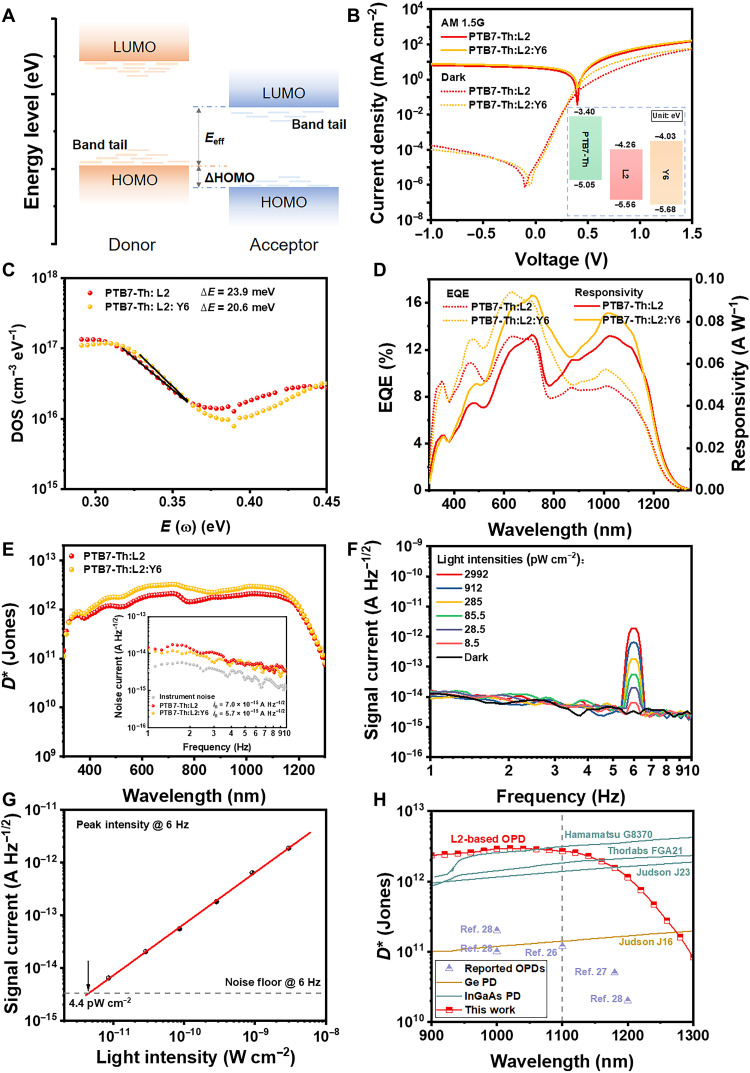
Optimization of NIR OPDs. (**A**) Schematic diagram of the bulk heterojunction structure showing the band alignment and the band tail states. (**B**) Characteristic *J-V* curves under AM 1.5G irradiation (100 mW cm^−2^) and in the dark (inset: energy levels of PTB7-Th, L2, and Y6 derived from UPS and LEIPS). (**C**) Trap DOS curves. The solid lines are linear fitting to the exponential bandtails, and the Δ*E* is the bandtail disorder spread. (**D**) EQE spectra and responsivity under zero bias. (**E**) Specific detectivity under zero bias (inset: the measured device noise current under zero bias). (**F**) The signal current of PTB7-Th:L2:Y6-based device under zero bias under 1-μm light illumination modulated at 6 Hz with various light intensities. (**G**) The peak signal intensity at 6 Hz obtained from (F) as a function of light intensities. The solid line is a linear fitting to the data, and the dashed line is the device noise level in the dark. (**H**) The summary of specific detectivity of reported NIR OPDs operating in photovoltaic mode with photoresponse beyond 1.1 μm and commercial PDs.

When OPDs operate at photovoltaic mode, the *i*_thermal_ dominates the total noise in our OPD device. According to the physical model developed by Ng *et al.* (*59*), higher effective bandgap (*E*_eff_) and lower band tail disorder spread (∆*E*) could lead to a smaller *i*_thermal_ (more details seen in the Supplementary Materials). *E*_eff_ is defined as the difference between the HOMO of the donor and the LUMO of the acceptor (Fig. 4A). As depicted in Fig. 4B and fig. S12, the EA of L2 was obtained as 4.26 eV through the measurement of low-energy inverse photoemission spectroscopy (LEIPS), and the ionization potential of PTB7-Th was evaluated as 5.05 eV (*60*) from ultraviolet photoelectron spectroscopy (UPS) result, which means that the LUMO of L2 is −4.26 eV and the HOMO of PTB7-Th is −5.05 eV (*61*). The obtained *E*_eff_ value of 0.79 eV for PTB7-Th:L2 is much smaller than the *E*_g_^opt^ of L2 (1.03 eV). The ∆*E* value was acquired via the measurement of capacitance versus frequency. According to the fitting of the exponential band tails, a very low ∆*E* value of 23.9 ± 1.1 meV was obtained (1/slope fitted in Fig. 4C), even smaller than thermal energy *k*_B_T at room temperature.

Considering that the ∆*E* (23.9 ± 1.1 meV) of PTB7-Th:L2 blend film has been among the smallest values for organic thin films (*59*), the small *E*_eff_ should play a crucial role in restricting the noise current of our OPD device. Thus, increasing *E*_eff_, that is, downshifting the HOMO of donor and/or upshifting the LUMO of acceptor, is the key for further decreasing the noise current. Meanwhile, it should be also considered that sizeable HOMO offset between the donor and acceptor is required for efficient exciton–to–charge transfer state conversion and high responsivity (*60*). The threshold HOMO offset originates from the vacuum level shift (ΔVL) induced by the collective electrostatic effects of the dipole layer, which can be calculated by (*61*)

$$\text{Δ}\mathrm{VL}=\frac{e\mu_{0}}{\varepsilon_{0}\varepsilon_{r}A_{o}}$$ (7)

where μ_0_ is the dipole moment in the designated y direction created by the isolated D-A unit pair (taking a positive value when the dipole points from A unit to D unit), *A*_o_ is the corresponding overlapped molecular area, ε_0_ and ε_r_ correspond to vacuum and relative permittivity, respectively. For PTB7-Th:L2 blend film, the μ_0_ is calculated as 1.0543 Debye, ε_r_ is obtained as 3.0 from the capacitance-frequency measurements, *A*_o_ is assumed as 20 Å^2^ (*61*), and then ΔVL can be acquired as 0.66 eV (figs. S13 and S14). According to the UPS result of L2 (HOMO = −5.56 eV), the HOMO offset between PTB7-Th and L2 is calculated as 0.51 eV, which is obviously lower than the ΔVL value and indicates that the charge separation is insufficient in PTB7-Th:L2–based devices. To verify the deduction, the measurement of the photocurrent density (*J*_ph_)–effective voltage (*V*_eff_) was carried out. As shown in fig. S15, the *J*_ph_ does not reach saturation even at a high *V*_eff_ of >4 V, and the exciton dissociation efficiency (*P*_diss_) is only 43% (detailed calculation process seen in the Supplementary Materials), which is also responsible for the low EQE value of <10% in the NIR region of our OPD devices at zero bias, besides the influence of energy gap law.

To further reduce the noise as well as the remaining efficient charge separation and high responsivity, the *E*_eff_ is required to be improved and the threshold HOMO offset, that is, the ΔVL value needs to be reduced for PTB7-Th:L2. One of the simple and feasible strategies is introducing a suitable third component. For a donor third component, it should exhibit deeper HOMO than that of PTB7-Th and much smaller μ_0_ with L2 relative to that of PTB7-Th:L2. For an acceptor third component, it should exhibit higher LUMO than that of L2 as well as small μ_0_ with PTB7-Th. High-performance acceptor material Y6 (*21*) exhibits higher LUMO energy of −4.03 eV (*62*) relative to L2 (Fig. 4B), and the μ_0_ of PTB7-Th:Y6 is calculated as 0.8775 Debye, which is notably lower than that (1.0543 Debye) of PTB7-Th:L2 (fig. S14). Moreover, it has been found that the addition of Y6 with good crystallinity could restrain the nonradiative recombination with improved charge transport and collection (*63*). Taking these into account, we introduced Y6 as the third component into PTB7-Th:L2 blend film. As shown in Fig. 4E, because of the higher *E*_eff_ and lower Δ*E* of 20.7 ± 0.5 meV (Fig. 4C), the device based on PTB7-Th:L2:Y6 shows a lower measured noise of 5.7 × 10^−15^ A Hz^−1/2^ (average value in the test frequency range) than that of PTB7-Th:L2–based one. Because of more efficient charge separation (*P*_diss_ = 46%; fig. S15), higher EQE intensity with a maximum responsivity of 0.084 A W^−1^ at λ = 1.02 μm in the NIR region is acquired (Fig. 4D). Consequently, in comparison with the PTB7-Th:L2–based one, increased D^*^ values above 10^12^ Jones can be obtained in the range of 0.41 to 1.2 μm, and a maximum value of 2.9 × 10^12^ Jones is achieved at the wavelength of 1.02 μm under zero bias for the device based on PTB7-Th:L2:Y6 (Fig. 4E). Under 1-μm-wavelength illumination, the PTB7-Th:L2:Y6 ternary OPDs at zero bias also show the linear response down to the weakest light we can obtain and accurately calibrate (8.5 pW cm^−2^) but stronger light response at the same intensity and lower linear-extrapolated value of 4.4 pW cm^−2^ (Fig. 4, F and G), relative to the PTB7-Th:L2 binary devices. It is worth noting that the calculated D^*^ values of our OPDs are one order higher than that of commercial Ge detector and even comparable to those of commercial InGaAs PDs in the wavelength range of 0.9 to 1.2 μm, despite the detection limit of our OPDs is shorter than Ge and InGaAs detectors (~1.7 μm) and D^*^ are still lower than the state-of-the-art InGaAs PDs in the long-wavelength region, like Hamamatsu G8370 (Fig. 4H) (*29*–*32*).

### Proof of concept for applications of NIR OPDs

**Fig. 5.**
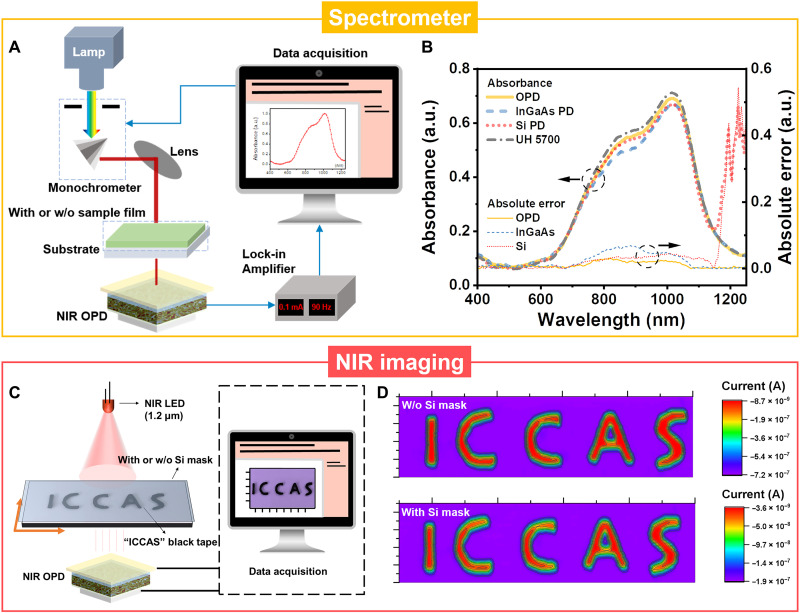
Proof of concept for applications of NIR OPDs. (**A**) Schematic diagram of the spectrometer prototype using the NIR OPD we developed as the detector. (**B**) Comparison of the absorption spectra measured by our NIR OPD, commercial InGaAs PD (Hamamatsu G8370-03), and Si PD (Hamamatsu S1337-1010BQ). The spectrum measured by commercial spectrometer (UH 5700, Hitachi) is used as the reference. The absolute error is defined as the absorbance difference between the PD and UH 5700. The sample is COTIC-4Cl film. (**C**) Schematic diagram of the optical imaging with or without Si mask. (**D**) Imaging of ICCAS letter graphics without (top) and with (down) Si mask under LED illumination (1.2 μm) at 0.72 mW cm^−2^.

As a powerful tool in various research areas, the commercial spectrometer with a spectral response beyond 1.1 μm must contain a Si photodiode and an additional NIR PD made of Ge or InGaAs, restricting its miniaturization and integration. In contrast, the emerging low-cost and light-weight NIR OPDs are among the most promising candidates for spectrometers with wide-range spectral response (*64*). Here, a spectrometer prototype was established using PTB7-Th:L2-based conventional OPD devices as the key element (Fig. 5A). Meanwhile, a commercial Si PD (Hamamatsu S1337-1010BQ) and an InGaAs PD (Hamamatsu G8370-03) were also used as the detector in the prototype for comparation of the measured spectra. The transmittance (*T*) and corresponding absorbance (Abs) of the sample film [here is COTIC-4Cl (*64*), chemical structure seen in fig. S16A] to different monochromatic light can be calculated by the following equations

$$T=\frac{I_{\mathrm{sample}}}{I_{\mathrm{blank}}}\times 100\%$$ (8)

$$\mathrm{Abs}=-\log\left(\frac{T}{100\%}\right)$$ (9)

where *I*_sample_ is the current signal of the OPD with the sample as the optical filter, and *I*_blank_ is the current signal of the OPD without a sample as the optical filter (fig. S16). As shown in Fig. 5B, the absorbance measured by our OPD is almost identical to that obtained by a commercial spectrometer UH 5700 (Hitachi High-Tech Science Corporation) due to the large LDR and high sensitivity of our OPD device. It should be also noted that small absolute errors of <0.03 still exist, which might be attributed to the differences of the measurement setup (e.g., light source, optical grating, and signal amplifier) (*64*). In contrast, the measured data of the Si PD become invalid beyond the wavelength of 1.13 μm due to the spectral response limit of Si. The spectrum measured by InGaAs PD shows clear deviation with absolute errors of ~0.06 relative to that of UH 5700 in the range of 0.8 to 0.9 μm, which may be caused by the weak light intensity of tungsten lamp (fig. S17) and the low sensitivity of InGaAs PD in this range besides the differences of the measurement setup (*32*). The results prove that the NIR OPD we developed can be used to construct a spectrometer with wide-range spectra response from 0.4 to 1.25 μm, which is approximately 120 nm beyond the limit of Si PDs.

Then, to expand the application of our OPD in NIR region beyond Si detector, a prototype experiment of single-pixel NIR optical imaging was carried out under 1.2-μm-wavelength light. “ICCAS,” which is the abbreviation of “Institute of Chemistry, Chinese Academy of Sciences”, is chosen as the object and patterned by black tape to block light (Fig. 5C), and the transmittance spectrum of black tape is shown in fig. S18. The object is mounted onto a computerized two-dimensional translational platform, which can be moved continuously in both *X* and *Y* directions on the horizontal plane. Under the illumination of light-emitting diode (LED; 1.2 μm), the NIR OPD can only receive the incident light passing through the transparent part of the mask, while the illumination on the object ICCAS would be absorbed by the black tape. As shown in Fig. 5D, the image for the object ICCAS exhibits clear boundaries. When a 300-μm-thick Si mask is covered on the whole scanning region including the black tape ICCAS and the transparent part, the obtained image remains distinguishable (Fig. 5D), although the optical response of the NIR OPD is reduced under the same incident light intensity due to the propagation loss (fig. S19). These results verify that our fabricated OPDs are promising to be applied in NIR imaging, especially for the incident light with energy below Si bandgap.

In summary, an efficient molecular design strategy to realize ultra-narrow bandgap is developed via introducing a cyano-contained quinoid end-capping group with enhanced electronegativity. The synthesized QC-based small-molecule semiconductors exhibit notably smaller bandgaps with ∆*E*_g_^opt^ of 0.4 to 0.45 eV than those of IC-based analogs. The obtained *E*_g_^opt^ value as low as 0.77 eV (with a large absorption onset at ~1.7 μm) in QC-based small molecules is the smallest *E*_g_^opt^ value reported for organic photovoltaic small-molecule semiconductors. The photodiode-type NIR OPDs based on our designed NIR-absorbing molecules exhibit high detectivity with a D^*^ value of up to 2.9 × 10^12^ Jones at 1.02 μm under zero bias, which is the highest value reported for NIR OPDs operating in photovoltaic mode with photoresponse below Si bandgap. The D^*^ values of our OPDs are even on a par with those of commercial InGaAs PDs in the wavelength range of 0.9 to 1.2 μm. The practical application potential of our NIR OPDs is verified well by the spectrometer prototype with a wide measurable region of 0.4 to 1.25 μm and the NIR imaging of the letters ICCAS under 1.2-μm-wavelength light, which demonstrates the commercialization potential of NIR OPDs we fabricated. The advances in this report will motivate the design of ultra-narrow bandgap semiconductors with high infrared detectivity and promote the development of other applications of NIR-sensitive organic semiconductors, such as semitransparent polymer solar cells, photoacoustic imaging, and photothermal therapy.

## MATERIALS AND METHODS

### Synthesis of QC-Br

To a solution of 2-bromonaphthalene-1,4-dione (1.78 g, 7.5 mmol) and malononitrile (500 mg, 7.5 mmol) in anhydrous dichloromethane (30 ml), 1 M titanium tetrachloride in anhydrous dichloromethane (7.5 ml, 7.5 mmol) was added dropwise under nitrogen at 0°C. After the mixture was stirred at 0°C for 0.5 hours, anhydrous pyridine (0.3 ml, 3.7 mmol) was added dropwise under nitrogen at 0°C. The mixture was stirred at 0°C for 0.5 hours. Water (30 ml) was added and the mixture was extracted with dichloromethane (2 × 30 ml). The organic phase was dried over anhydrous MgSO_4_ and filtered. After removing the solvent from the filtrate, the residue was purified by column chromatography on silica gel using petroleum ether/ethyl acetate (25:1) as the eluent yielding a yellow solid (360 mg, 17%). ^1^H nuclear magnetic resonance (NMR) (400 MHz, CDCl_3_): δ 8.82 (m, 1H), 8.36 (m, 2H), 7.83 (m, 2H). ^13^C NMR (100 MHz, CDCl_3_): δ 176.42, 152.67, 138.25, 135.19, 134.47, 134.31, 130.14, 129.70, 129.40, 126.98, 113.73, 112.60, 84.83. Electron-impact mass spectrometry: *m/z* 283.9 (M^+^).

### Inverted device fabrication and characterization

The inverted structure of OPD devices was ITO/ZnO/active layer/MoO_3_/Ag. Patterned ITO glass (sheet resistance = 15 ohm) was precleaned in an ultrasonic bath with ultra-pure water, acetone, and isopropanol and treated in an ultraviolet-ozone chamber (Mondel UV-03 UVO_3_ cleaner) for 20 min. ZnO layer (ca. 30 nm) was spin-coated at 4000 rpm onto the ITO glass from ZnO precursor solution [100 mg of Zn(CH_3_COO)_2_·2H_2_O and 28.29 μl of ethanolamine dissolved in 973 μl of 2-methoxyethanol] and then baked at 200°C for 30 min. PTB7-Th:L2 (1:1.2, w/w) blends were dissolved in CHCl_3_ containing 0.2 volume % 1-chloronaphthalene (1-CN) (15 mg ml^−1^ in total) and then spin-coated onto ZnO layer at 2000 rpm for 40 s followed by thermal annealing at 110°C for 5 min to form the photoactive layers (128 ± 5 nm) in nitrogen glove box. PDPPDTP: L4 (1:1, w/w) blends were dissolved in CHCl_3_ (8 mg ml^−1^ in total) and then spin-coated onto ZnO layer at 1600 rpm for 40 s followed by thermal annealing at 130°C for 5 min to form the photoactive layers (100 ± 3 nm) in nitrogen glove box. PTB7-Th:L2:Y6 (1:0.9:0.3, w/w) blends were dissolved in CHCl_3_ containing 0.2 volume % 1-CN (15 mg ml^−1^ in total) and then spin-coated onto ZnO layer at 2000 rpm for 40 s followed by thermal annealing at 110°C for 5 min to form the photoactive layers (132 ± 4 nm) in nitrogen glove box. The MoO_3_ layer (ca. 5 nm) and the Ag electrode (ca. 80 nm) were slowly evaporated onto the surface of the photoactive layer under vacuum (ca. 10^−5^ Pa). The active area of the device was ca. 0.04 cm^2^. The devices were not masked and the active area of devices was measured by optical microscopy. The *J-V* characteristics were measured under AM 1.5G illumination at 100 mW cm^−2^ using a solar simulator (Enlitech model SS-F5-3A) calibrated with a monocrystal Si reference cell equipped with a KG5 filter (certificated by the National Institute of Metrology) and a Keithley 2450 source measure unit. The dark current density-voltage characteristics were measured in the dark using a B2912A Precision Source/Measure Unit (Agilent Technologies). The test environment at home laboratory is 25° ± 2.5°C in a glove box. The EQE spectra and corresponding responsivity were measured using a solar cell spectral response measurement system (QE-R3011, Enli Technology Co. Ltd.). The light intensity at each wavelength was calibrated using a standard single crystal Si photovoltaic cell. The fabrication of conventional OPD device can be seen in the Supplementary Materials.

### Noise current measurement

The noise measurement of the OPD was conducted using a battery-powered preamplifier (Stanford SR570) coupled with a fast Fourier transform (FFT) signal analyzer (Agilent 35670A) in the dark. The preamplifer and device for test are put in a shielded metal box to avoid the electromagnetic interference from ambient. The encapsulated OPD device is connected to the input end of the Stanford SR570 preamplifier through the bayonet nut connector (BNC) cable. The connection between the output end of the preamplifier and the input end of the FFT signal analyzer also uses the BNC cable. The noise current of the device is converted into an amplified voltage signal by the preamplifier and then recorded by the FFT signal analyzer, which reports a voltage noise normalized to the measurement bandwidth in V Hz^−1/2^. The sensitivity of the preamplifier is adjusted to make sure that noise of device is obviously higher than the instrument noise floor. The actual noise current is calculated by dividing the voltage noise signal acquired from the FFT analyzer with the amplification ratio of the preamplifier. The reverse bias was applied by a multimeter.

### Noise equivalent power measurement

The noise equivalent power of the OPD was measured in the same as the noise measurement with a 1-μm LED modulated at the frequency of 6 Hz as the light source by a function generator, and a series of neutral density filters were used to attenuate its light intensities that were calibrated with the commercial Si photodiode (Hamamatsu S1133-01). It is expected that a peak at the frequency of 6 Hz will appear in the current spectrum when turning on the LED, and its peak intensity should be proportional to the photocurrent of the device. According to the definition of NEP, it represents the lowest light intensity under which the photocurrent can no longer be differentiated from the noise current. As a result, when the light intensity is decreased to a value that the peak at 6 Hz is merged into the background noise current, this light intensity can be considered as the NEP of the device.

### LDR measurement

A set of 1-μm LED was used as the light source, and a series of calibrated neutral density filters were used to modulate the incident light intensity. The light intensity was carefully calibrated with commercial Si photodiode (Hamamatsu S1133-01). The photocurrent of the device under different light intensities was recorded with a lock-in amplifier (SR 830).

### Spectrometer prototype

The whole system is composed of light source, monochromator, lens, detector, lock-in amplifier, and computer. The detector is PTB7-Th:L2–based conventional OPD device, while a commercial Si PD (Hamamatsu S1337-1010BQ) and an InGaAs PD (Hamamatsu G8370-03) are also used as the detector for comparation. Compound light is emitted by a tungsten lamp (CT-TH-150) and becomes monochromatic light after passing through a monochromator (CT-QEM-24). The monochromatic light is focused on the surface of the detector by lens, and a lock-in amplifier (SRS-SR830) is used to connect the detector to read the current. A computer is connected to both the lock-in amplifier and the monochromator to detect the photogenerated current under different monochromatic light irradiation. Through dividing the current with/without the sample film on the detector surface, we can obtain the transmittance and corresponding absorbance of the sample film to different monochromatic light. The luminescence spectrum of the tungsten lamp was measured by a commercial optical power meter (843-R, Newport). The sample is COTIC-4Cl film, which is formed by spin coating its CHCl_3_ solution (20 mg ml^−1^) at 2000 rpm for 30 s on a quartz substrate.

### Optical imaging

The single pixel imaging was performed by scanning a self-made mask using black tape (3M 1500) with letters of ICCAS (the size is 8 mm by 35 mm; the line width is 1 mm) along the *X* and *Y* directions with a motorized *X*-*Y* linear stage (PI M-406.2DG) under the excitation light intensity of 0.72 mW cm^−2^ for the 1.2-μm light source (Thorlabs LED 1200E). The digital source meter (Keithley 2400) is used to record the photoresponse signals at each position for imaging.

## Supplementary Materials

### This PDF file includes:

Supplementary Text Figs. S1 to S19 Tables S1 to S4 References
