# Supplementary material for: Sensitive photodetection below silicon bandgap using quinoid-capped organic semiconductors
Source: Sci Adv. 2023 Mar 29;9(13):eadf6152. doi: 10.1126/sciadv.adf6152 (PMC10058242; doi:10.1126/sciadv.adf6152)
Supplement: Supplementary file 1 — Supplementary Text Figs. S1 to S19 Tables S1 to S4 References [file sciadv.adf6152_sm.pdf]

Science Advances
AAAS

Supplementary Materials for

Sensitive photodetection below silicon bandgap using quinoid-capped organic semiconductors

Tengfei Li *et al.*

Corresponding author: Yuze Lin, linyz@iccas.ac.cn; Liang Shen, shenliang@jlu.edu.cn; Yanjun Fang, jkfang@zju.edu.cn

Sci. Adv. **9**, eadf6152 (2023)
DOI: 10.1126/sciadv.adf6152

This PDF file includes:

Supplementary Text
Figs. S1 to S19
Tables S1 to S4
References

Supplementary Text

Materials

Unless stated otherwise, all the solvents and chemical reagents used were obtained commercially and used without further purification. Toluene was distilled from sodium benzophenone under nitrogen before use. Compound 2, compound 3, IDIC, 6TIC, COT-CHO, and PDPPDTP were synthesized according to the literature procedure. (*25, 38, 40, 47, 65, 66*) IDT-2SnMe_3, Y5, PBDB-T, PM6 and Y6 were purchased from Solarmer Material Inc.; 2-bromonaphthalene-1,4-dione was purchased from Ark Pharm, Inc.; malononitrile was purchased from Innochem Science & Technology Co., Ltd.; BTP was purchased from Hyper, Inc.; 3TT was purchased from Suna Tech Inc.; DPO was purchased from Prui Material Technology Co., Ltd.; PTB7-Th was purchased from 1-Material Inc.

Synthesis

COTIC. To a three-necked round bottom flask were added COT-CHO (176 mg, 0.2 mmol), IC (155 mg, 0.8 mmol), pyridine (0.5 mL) and chloroform (30 mL). The mixture was deoxygenated with nitrogen for 15 min and then stirred at reflux for 12 h. After cooling to room temperature, the mixture was poured into methanol (200 mL) and filtered. The residue was purified by column chromatography on silica gel using petroleum ether/dichloromethane (1:1) as eluent yielding a purple solid (190 mg, 77%). ^{1}H NMR (300 MHz, $CDCl_3$): δ 8.73 (s, 2H), 8.66 (m, 2H), 7.89 (m, 2H), 7.72 (m, 4H), 7.62 (s, 2H), 7.48 (s, 2H), 4.16 (d, J = 5.2 Hz, 4H), 2.03-1.84 (m, 6H), 1.72-1.54 (m, 10H), 1.42 (m, 8H), 1.09-0.90 (m, 28H), 0.70 (m, 12H). ^{13}C NMR (75 MHz, $CDCl_3$): δ 188.58, 160.82, 160.20, 154.52, 142.55, 140.10, 136.74, 136.64, 136.60, 136.32, 134.86, 134.15, 131.82, 130.86, 129.41, 128.79, 125.08, 123.37, 122.09, 121.54, 115.06, 114.94, 74.68, 53.81, 43.48, 39.70, 35.27, 33.97, 30.52, 29.03, 28.40, 27.23, 23.92, 22.97, 22.77, 14.08, 13.94, 11.17, 10.48. MS (MALDI-TOF): *m/z* 1231.5 (M^+).

Compound 1. To a three-necked round bottom flask were added IDT-2SnMe$_3$ (186mg, 0.2 mmol), 2-bromonaphthalene-1,4-dione (100 mg, 0.42 mmol) and toluene (20 mL). The mixture was deoxygenated with nitrogen for 15 min. Pd(PPh$_3$)$_4$ (23 mg, 0.02 mmol) was added under nitrogen. The mixture was refluxed for 24 h and then cooled down to room temperature. Water (30 mL) was added and the mixture was extracted with dichloromethane (2 × 30 mL). The organic phase was dried over anhydrous MgSO$_4$ and filtered. After removing the solvent from the filtrate, the residue was purified by column chromatography on silica gel using petroleum ether/dichloromethane (2:1) as the eluent yielding a purple solid (121 mg, 66%). ^{1}H NMR (300 MHz, CD$_2$Cl$_2$): δ 8.17 (m, 2H), 8.06 (m, 2H), 7.81 (s, 2H), 7.78 (m, 4H), 7.48 (s, 2H), 7.28 (s, 2H), 2.10 (m, 4H), 1.95 (m, 4H), 1.10 (m, 24H), 0.87 (m, 8H), 0.75 (m, 12H). MS (MALDI-TOF): *m/z* 915.7 (M$^+$).

L1. To a solution of compound 1 (92 mg, 0.1 mmol) and malononitrile (40 mg, 0.6 mmol) in anhydrous dichloromethane (20 mL), 1 M titanium tetrachloride in anhydrous dichloromethane (0.6 mL, 0.6 mmol) was added dropwise under nitrogen at room temperature. After the mixture was stirred at room temperature for 0.5 h, anhydrous pyridine (0.1 mL, 1.2 mmol) was added dropwise under nitrogen at room temperature. The mixture was stirred at room temperature for 1 h. Water (30 mL) was added and the mixture was extracted with dichloromethane (2 × 30 mL). The organic phase was dried over anhydrous MgSO$_4$ and filtered. After removing the solvent from the filtrate, the residue was purified by column chromatography on silica gel using petroleum ether/dichloromethane (2:1) as the eluent yielding a green solid (90 mg, 89%). ^{1}H NMR (500 MHz, CDCl$_3$): δ 8.85 (m, 2H), 8.38 (m, 2H), 8.13 (s, 2H), 7.86 (s, 2H), 7.80 (m, 4H), 7.45 (s, 2H), 2.08 (m, 4H), 1.96 (m, 4H), 1.32-1.21 (m, 24H), 0.90-0.82 (m, 8H), 0.81-0.74 (m, 12H). ^{13}C NMR (75 MHz, CDCl$_3$) δ 182.54, 157.65, 155.30, 153.51, 152.67, 138.05, 137.38, 136.58, 134.02, 133.51, 130.61, 129.82, 128.95, 126.18, 125.41, 125.14,

115.21, 114.83, 114.13, 79.93, 76.61, 54.42, 39.23, 31.56, 29.57, 22.55, 14.00. MS (MALDI-TOF): *m/z* 1011.5 (M^+).

BTP-2SnMe$_3$. To a solution of BTP (194 mg, 0.2 mmol) in THF (20 mL) at −78 ºC was added 2 M LDA in THF (0.25 mL, 0.5 mmol) dropwise under nitrogen. The mixture was stirred at −78 ºC for 1.5 h, and then 1 M trimethyltin chloride in THF (0.5 mL, 0.5 mmol) was added. The mixture was stirred overnight at room temperature. Saturated KF aqueous solution (30 mL) was added and the mixture was extracted with dichloromethane (2 × 30 mL). The organic phase was dried over anhydrous $MgSO_4$ and filtered. After removing the solvent from the filtrate, the residue was poured into methanol (100 mL) and filtered yielding an orange solid (220 mg, 85%). ^{1}H NMR (400 MHz, $CDCl_3$): δ 4.62 (m, 4H), 2.82 (d, J = 8.0 Hz, 4H), 2.05 (m, 2H), 1.84 (m, 4H), 1.47-1.23 (m, 36H), 1.02-0.83 (m, 18H), 0.66-0.57 (m, 12H), 0.49 (s, 18H).

L2. To a three-necked round bottom flask were added BTP-2SnMe$_3$ (169 mg, 0.13 mmol), QC-Br (74 mg, 0.26 mmol) and toluene (20 mL). The mixture was deoxygenated with nitrogen for 15 min. $Pd(PPh_3)_4$ (15 mg, 0.013 mmol) was added under nitrogen. The mixture was refluxed for 6 h and then cooled down to room temperature. Water (30 mL) was added and the mixture was extracted with dichloromethane (2 × 30 mL). The organic phase was dried over anhydrous $MgSO_4$ and filtered. After removing the solvent from the filtrate, the residue was purified by column chromatography on silica gel using petroleum ether/ethyl acetate (25:1) as the eluent yielding a black solid (97 mg, 54%). ^{1}H NMR (300 MHz, $CDCl_3$): δ 8.84 (m, 2H), 8.38 (m, 2H), 7.81 (s, 2H), 7.83 (m, 4H), 4.69 (d, J = 6.0 Hz, 4H), 3.09 (t, J = 9.0 Hz, 4H), 2.14-1.94 (m, 6H), 1.46-1.21 (m, 28H), 1.14-0.80 (m, 16H), 0.74-0.60 (m, 12H). ^{13}C NMR (100 MHz, $CDCl_3$): δ 183.05, 152.29, 147.57, 145.71, 141.45, 137.62, 137.03, 134.02, 133.58, 132.61, 132.59, 130.44, 130.33, 129.69, 129.01, 128.97, 128.93, 126.17, 126.10,

115.02, 113.87, 112.70, 81.11, 65.38, 55.55, 42.06, 40.23, 31.99, 31.03, 30.22, 30.07, 29.80, 29.74, 29.71, 29.69, 29.50, 29.42, 29.19, 29.15, 27.78, 23.44, 23.31, 23.15, 22.88, 22.76, 14.18, 14.14, 13.81, 11.17, 10.31. MS (MALDI-TOF): *m/z* 1379.1 (M^+).

3TT-2SnMe$_3$. To a solution of 3TT (216 mg, 0.2 mmol) in THF (20 mL) at −78 ºC was added 1.6 M *n*-butyllithium in hexane (0.32 mL, 0.5 mmol) dropwise under nitrogen. The mixture was stirred at −78 ºC for 1.5 h, and then 1 M trimethyltin chloride in THF (0.5 mL, 0.5 mmol) was added. The mixture was stirred overnight at room temperature. Saturated KF aqueous solution (30 mL) was added and the mixture was extracted with dichloromethane (2 × 30 mL). The organic phase was dried over anhydrous $MgSO_4$ and filtered. After removing the solvent from the filtrate, the residue was poured into methanol (100 mL) and filtered yielding an orange solid (253 mg, 90%). ^{1}H NMR (400 MHz, CD_2Cl_2): δ 7.30 (s, 2H), 7.10 (d, J = 8.4 Hz, 8H), 7.08 (d, J = 8.4 Hz, 8H), 2.53 (t, J = 8.0 Hz, 8H), 1.54 (m, 8H), 1.33-1.22 (m, 24H), 0.83 (m, 12H), 0.36 (s, 18H).

L3. To a three-necked round bottom flask were added 3TT-2SnMe$_3$ (141 mg, 0.1 mmol), QC-Br (68 mg, 0.24 mmol) and toluene (20 mL). The mixture was deoxygenated with nitrogen for 15 min. $Pd(PPh_3)_4$ (12 mg, 0.01 mmol) was added under nitrogen. The mixture was refluxed for 11 h and then cooled down to room temperature. Water (30 mL) was added and the mixture was extracted with dichloromethane (2 × 30 mL). The organic phase was dried over anhydrous $MgSO_4$ and filtered. After removing the solvent from the filtrate, the residue was purified by column chromatography on silica gel using petroleum ether/dichloromethane (3:1) as the eluent yielding a brown solid (91 mg, 61%). ^{1}H NMR (300 MHz, $CDCl_3$): δ 8.82 (m, 2H), 8.32 (m, 2H), 8.24 (s, 2H), 8.02 (s, 2H), 7.77 (m, 4H), 7.16 (m, 16H), 2.57 (t, J = 8.1 Hz, 8H), 1.63-1.54 (m, 8H), 1.36-1.22 (m, 24H), 0.90-0.82 (m, 12H). ^{13}C NMR (75 MHz, $CDCl_3$) δ 182.43, 152.12, 151.16, 148.44, 145.28, 143.29, 142.82, 141.77, 139.27,

137.35, 137.04, 136.27, 134.13, 133.51, 130.57, 129.92, 129.15, 128.95, 127.89, 126.25, 125.59, 124.87, 62.44, 35.74, 31.78, 31.30, 29.28, 22.68, 14.18. MS (MALDI-TOF): *m/z* 1490.1 (M^+).

COT. To a three-necked round bottom flask were added compound 2 (146 mg, 0.2 mmol), compound 3 (128 mg, 0.44 mmol) and toluene (20 mL). The mixture was deoxygenated with nitrogen for 15 min. $Pd(PPh_3)_4$ (25 mg, 0.022 mmol) was added under nitrogen. The mixture was refluxed for 12 h and then cooled down to room temperature. Water (30 mL) was added and the mixture was extracted with dichloromethane (2 × 30 mL). The organic phase was dried over anhydrous $MgSO_4$ and filtered. After removing the solvent from the filtrate, the residue was purified by column chromatography on silica gel using petroleum ether as the eluent yielding an orange oil (53 mg, 32%). ^{1}H NMR (300 MHz, $CDCl_3$): δ 7.08 (s, 2H), 7.00 (d, J = 5.4 Hz, 2H), 6.86 (d, J = 5.4 Hz, 2H), 4.04 (d, J = 6.0 Hz, 4H), 1.96-1.76 (m, 6H), 1.70-1.47 (m, 10H), 1.39 (m, 8H), 1.11-0.90 (m, 28H), 0.83-0.71 (m, 6H), 0.70-0.60 (m, 6H). ^{13}C NMR (75 MHz, $CDCl_3$): δ 156.94, 152.19, 136.05, 134.78, 120.34, 117.78, 117.39, 116.57, 74.09, 53.57, 43.44, 40.01, 35.21, 34.21, 30.63, 29.25, 28.67, 27.46, 24.01, 23.19, 22.96, 14.26, 14.17, 11.32, 10.74. MS (MALDI-TOF): *m/z* 822.5 (M^+).

COT-2SnMe$_3$. To a solution of COT (164 mg, 0.2 mmol) in THF (20 mL) at −78 ºC was added 2 M LDA in THF (0.25 mL, 0.5 mmol) dropwise under nitrogen. The mixture was stirred at −78 ºC for 1.5 h, and then 1 M trimethyltin chloride in THF (0.5 mL, 0.5 mmol) was added. The mixture was stirred overnight at room temperature. Saturated KF aqueous solution (30 mL) was added and the mixture was extracted with dichloromethane (2 × 30 mL). The organic phase was dried over anhydrous $MgSO_4$ and filtered. After removing the solvent from the filtrate, the residue was poured into methanol (100 mL) and filtered yielding an orange solid (218 mg, 95%). The product was directly used for next step reaction without further purification due to poor stability in air.

L4. To a three-necked round bottom flask were added COT-2SnMe$_3$ (115 mg, 0.1 mmol), QC-Br (68 mg, 0.24 mmol) and toluene (20 mL). The mixture was deoxygenated with nitrogen for 15 min. Pd(PPh$_3$)$_4$ (12 mg, 0.01 mmol) was added under nitrogen. The mixture was refluxed for 6 h and then cooled down to room temperature. Water (30 mL) was added and the mixture was extracted with dichloromethane (2 × 30 mL). The organic phase was dried over anhydrous MgSO$_4$ and filtered. After removing the solvent from the filtrate, the residue was purified by column chromatography on silica gel using petroleum ether/ethyl acetate (50:1) as the eluent yielding a brown solid (64 mg, 52%). ^{1}H NMR (400 MHz, CDCl$_3$): δ 8.81 (d, J = 8.0 Hz, 2H), 8.33 (m, 2H), 7.90 (t, J = 3.2 Hz, 2H), 7.75 (p, J = 7.2 Hz, 4H), 7.60 (t, J = 4.0 Hz, 2H), 7.44 (t, J = 3.2 Hz, 2H), 4.18 (d, J = 5.2 Hz, 4H), 2.06-1.84 (m, 6H), 1.72-1.50 (m, 10H), 1.42 (m, 8H), 1.08-0.91 (m, 28H), 0.73 (m, 6H), 0.67 (m, 6H). ^{13}C NMR (100 MHz, CDCl$_3$): δ 182.80, 160.44, 155.36, 151.16, 140.82, 136.86, 133.98, 133.92, 133.10, 130.45, 130.13, 130.05, 128.74, 126.07, 124.00, 120.56, 118.25, 115.63, 114.56, 74.57, 53.77, 43.45, 39.88, 35.38, 34.14, 30.62, 29.17, 28.52, 27.38, 24.02, 23.03, 22.85, 14.12, 14.01, 11.31, 10.59. MS (MALDI-TOF): m/z 1231.4 (M$^+$).

General characterization

The ^{1}H and ^{13}C NMR spectra were obtained using Bruker AVANCE (300 MHz, 400 MHz, and 500 MHz) spectrometer. Mass spectra were recorded using APEX II FT-ICR Mass Spectrometer (Bruker Daltonics, Inc.) and Autoflex III MALDI-TOF Mass Spectrometer (Bruker Daltonics, Inc.). UV-vis-NIR absorption spectra (solution in CHCl$_3$; thin film on quartz substrate) were measured using UH 4150 or UH 5700 spectrophotometer (Hitachi High-Tech Science Corporation). The electrochemical properties were investigated in deoxygenated anhydrous acetonitrile under nitrogen at a scan rate of 100 mV s^{-1} using 0.1 M tetrakis(n-butyl)ammonium hexafluorophosphate [(n-

$Bu_4)N^+PF_6^-$] as supporting electrolyte using a computer-controlled CHI660C electrochemical workstation, a glassy carbon working electrode, a platinum-wire auxiliary electrode, and an Ag/AgCl reference electrode. Films were drop-cast onto the working electrode from a 10 mg mL^{-1} $CHCl_3$ solution. A ferrocene/ferrocenium ($FeCp_2^{0/+}$) redox couple was used as internal standard and was assigned an absolute energy of –5.1 eV vs vacuum. Thermogravimetric analysis (TGA) was measured with a Shimadzu DTG 60 instrument from 50 to 500 °C at a heating rate of 10 °C min^{-1} under nitrogen atmosphere. Ultraviolet photoelectron spectroscopy (UPS) spectra were obtained from AXIS ULTRA DLD (Kratos) with He I (21.22 eV) excitation lines and a sample bias of –9 V under a vacuum of 3.0 × 10^{-8} Torr. Low-energy inverse photoemission spectroscopy (LEIPS) measurement was performed on a customized ULVAC-PHI LEIPS instrument with Bremsstrahlung isochromatic mode.

Conventional Device fabrication

The conventional structure of OPD devices was ITO/PEDOT:PSS/active layer/DPO/Al. PEDOT:PSS layer (*ca.* 30 nm) (Heraeus Clevios P VP. AI 4083, filtered at 0.45 μm) was spin-coated at 4000 rpm onto the ITO glass and then baked at 150 °C for 15 min. PM6: L1 (1:1.2, w/w) blends were dissolved in $CHCl_3$ containing 0.2 vol.% 1,8-diiodooctane (DIO) (13 mg mL^{-1} in total) and then spin-coated onto PEDOT:PSS layer at 2000 rpm for 40 s followed by thermal annealing at 110 °C for 5 min to form the photoactive layers (98±3 nm) in nitrogen glove box. PTB7-Th: L2 (1:1.2, w/w) blends were dissolved in $CHCl_3$ containing 0.2 vol.% 1-chloronaphthalene (1-CN) (15 mg mL^{-1} in total) and then spin-coated onto PEDOT:PSS layer at 2000 rpm for 40 s followed by thermal annealing at 110 °C for 5 min to form the photoactive layers (138±5 nm) in nitrogen glove box. PTB7-Th: L3 (1:1.2, w/w) blends were dissolved in $CHCl_3$ containing 0.2 vol.% 1-CN (15 mg mL^{-1} in total) and then spin-coated onto PEDOT:PSS layer at 2000 rpm for 40 s followed by thermal annealing at 110 °C

for 5 min to form the photoactive layers (136±4 nm) in nitrogen glove box. PDPPDTP: L4 (1:1, w/w) blends were dissolved in $CHCl_3$ (8 mg mL^{-1} in total) and then spin-coated onto PEDOT:PSS layer at 1600 rpm for 40 s followed by thermal annealing at 130 °C for 5 min to form the photoactive layers (102±3 nm) in nitrogen glove box. The active layer of IC-based analogues (IDIC, Y5, 6TIC, and COTIC) were fabricated through the same process as L1-L4. The DPO solution (0.5 mg mL^{-1} in isopropanol) was spin-coated on the active layer at 2000 rpm for 30 s. Finally, Al electrode (*ca*. 60 nm) was slowly evaporated onto the surface of the photoactive layer under vacuum (*ca*. 10^{-5} Pa).

Grazing-incidence wide-angle X-ray scattering measurement

GIWAXS measurement was performed at beamline 7.3.3 (*42*) at the Advanced Light Source. Samples were prepared by spin-coating L2 or Y5 solution (16 mg mL^{-1} in $CHCl_3$) at 2000 rpm for 30 s on Si substrates. The 10 keV X-ray beam was incident at a grazing angle of 0.11°-0.15°, selected to maximize the scattering intensity from the samples. The scattered X-rays were detected using a Dectris Pilatus 2M photon counting detector.

Molecular modelling

Density functional theory (DFT) calculations were performed with the Gaussian 16 program, using the B3LYP functional. (*67*) All-electron double-ξ valence basis sets with polarization functions 6-31G(d,p) were used for all atoms. Geometry optimizations were performed with full relaxation of all atoms in gas phase without solvent effects. Vibration frequency calculation was performed to check that the stable structures had no imaginary frequency. The calculations of Mulliken electronegativity, TDOS, PDOS, and electrostatic potential (ESP) distribution were carried out by a wavefunction analysis tool Multiwfn. (*68*)

Physical model of $i_{\mathrm{thermal}}(E_{\mathrm{eff}}, \Delta E)$

When OPDs operate at photovoltaic mode (0 V bias), the i_{thermal} dominates the total noise in our OPD device but the dark current could not make a contribution. According to the study by Ng. et al., the i_{thermal} is proportional to thermally generated intrinsic carrier concentration N_{i}. (*59*) As charge carrier transport in organic semiconductors is thermally activated, the carriers located deep in the bandgap (sub-gap states), have a small probability of being thermally excited, which would not obviously contribute to the N_{i} value and further the i_{thermal}. (*69*) In contrast, the carriers residing in bandtail trap are more likely to get thermally excited back into the band which can significantly affect the N_{i} and further the i_{thermal}. A general physical model was developed by Ng. et al. correlating i_{thermal} and the effective bandgap (E_{eff}) and band tail disorder spread (ΔE) in organic semiconductors. (*59*) E_{eff} is defined as the difference between the HOMO of the donor and the LUMO of the acceptor. The dependence of i_{thermal} on E_{eff} and ΔE can be expressed by the following equations:

$$i_{thermal} = C \cdot N_i$$

$$N_i = \int_0^{\infty} DOS(E) \cdot F(E) d(E) = \int_0^{\infty} DOS(E) \cdot \frac{1}{e^{\frac{E-E_F}{k_B T}} + 1} d(E)$$

$$DOS(E) = N_C \cdot e^{-\frac{\frac{E_{eff}}{2} - E}{\Delta E}}, \text{ for } E < \frac{E_{eff}}{2}$$

$$DOS(E) = \frac{N_C}{\sqrt{\frac{E_{eff}}{2}}} \cdot \sqrt{E} \text{ , for } E \geq \frac{E_{eff}}{2}$$

where C is the scaling factor, $F(E)$ is the Fermi–Dirac distribution, E_{F} is the fermi level, and N_{C} is the effective DOS at the band edge. The boundary condition is $\Delta E < \frac{E_{eff}}{13.8}$. The third equation accounts for the exponential distribution of bandtail states below the band edge. The fourth equation avoids discontinuity at the band edge and follows the conventional $\sqrt{E}$ dependence for energies above the band edge.

Dielectric constant measurement

The dielectric constant was determined by the parallel-plate-capacitance measurement with a device structure of ITO/PEDOT:PSS/test film/Ca/Al. A thin layer (30 nm) of PEDOT:PSS was spin-coated at 4000 rpm onto the ITO glass and then baked at 150 °C for 15 min. The blend film samples were formed through the same preparation condition as the photovoltaic devices. Then, the Ca layer (*ca.* 20 nm) and Al layer (*ca.* 60 nm) were subsequently evaporated onto the surface of the test film under vacuum (*ca.* 10^{-5} Pa). The measurement was performed using a Keysight 4980A LCR meter by sweeping the frequency. The ε_r value can be obtained from the equation:

$$C = \varepsilon_0 \varepsilon_r A/d$$

where C is the measured capacitance, ε_0 is the permittivity of free space, A is the device area, and d is the thickness of the test film.

Trap DOS measurements

Trap DOS were performed on Keysight 4980A LCR meter and analyzed by using the thermal admittance spectroscopy method. Trap DOS can be derived from angular frequency dependent capacitance according to the following formula:

$$N_{\mathrm{t}}(E_\omega) = -\frac{V_{\mathrm{bi}}}{eW}\frac{dC}{d\omega}\frac{\omega}{k_B T}$$

where V_{bi} and W are the built-in potential and depletion width, respectively, which were extracted from Mott-Schottky analysis. e is the elementary charge, C is the capacitance, k_B is Boltzmann constant, and T is the temperature. ω is the angular frequency defined by the following formula:

$$E_\omega = k_B \mathrm{T} \ln\left(\frac{\omega_0}{\omega}\right)$$

where ω_0 is the attempt-to-escape frequency. (*70*)

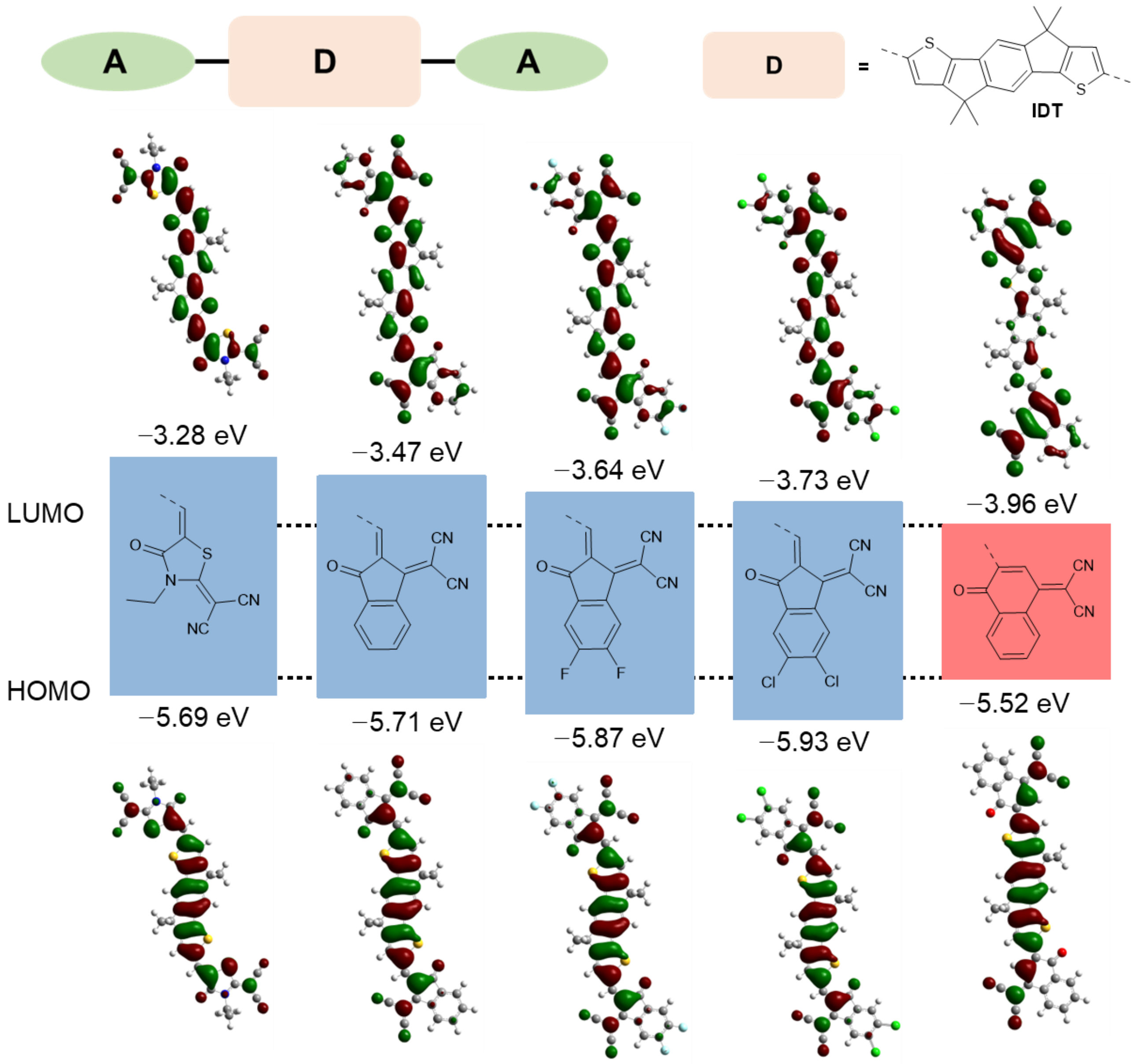

Fig. S1. Energy levels and electron distributions of frontier orbitals of IDT-based A-D-A molecules with QC and other widely-used "A" groups calculated by Gaussian 16 program at B3LYP/6-31G(d,p) level.

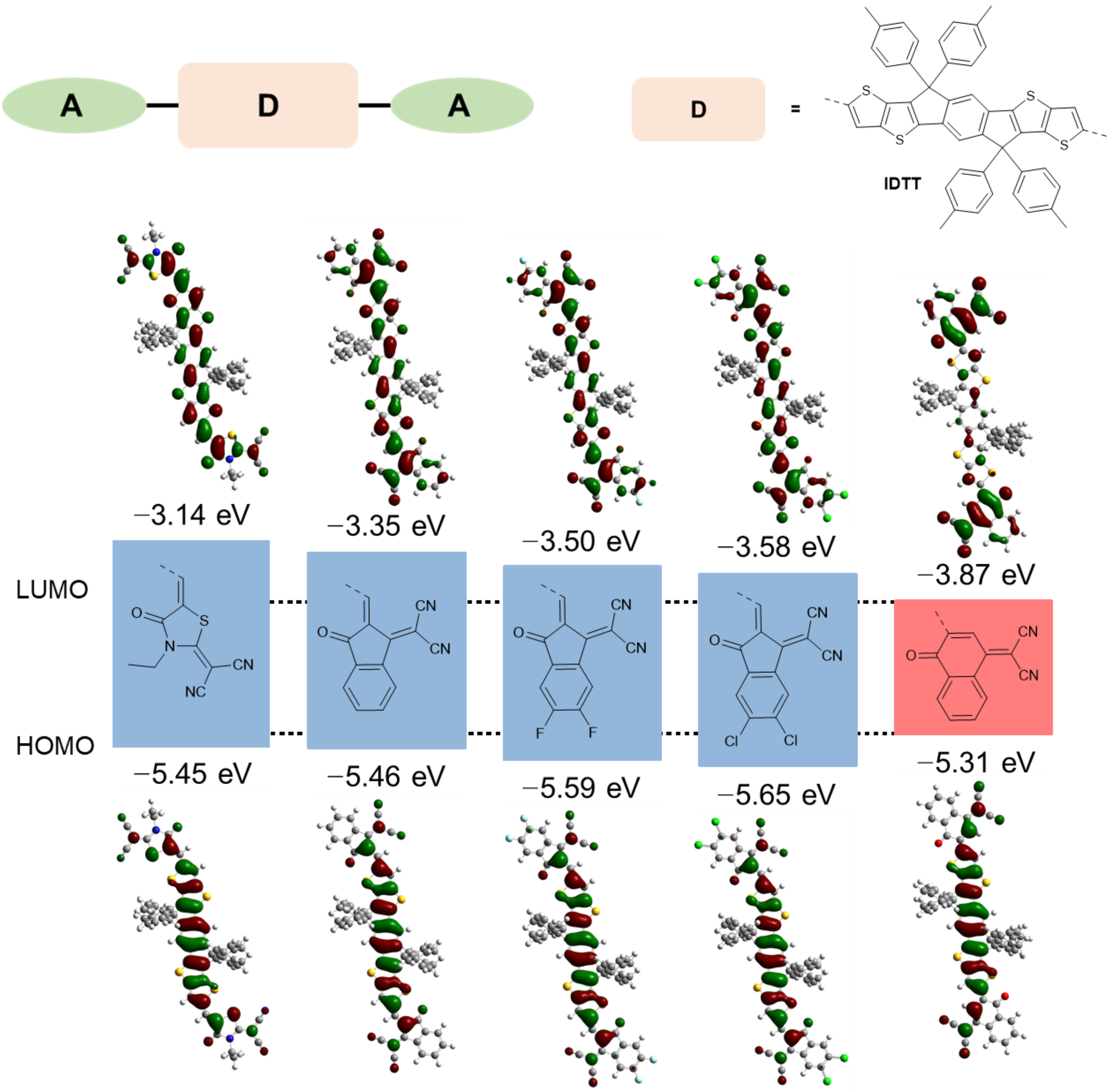

Fig. S2. Energy levels and electron distributions of frontier orbitals of IDTT-based A-D-A molecules with different "A" groups calculated by Gaussian 16 program at B3LYP/6-31G(d,p) level.

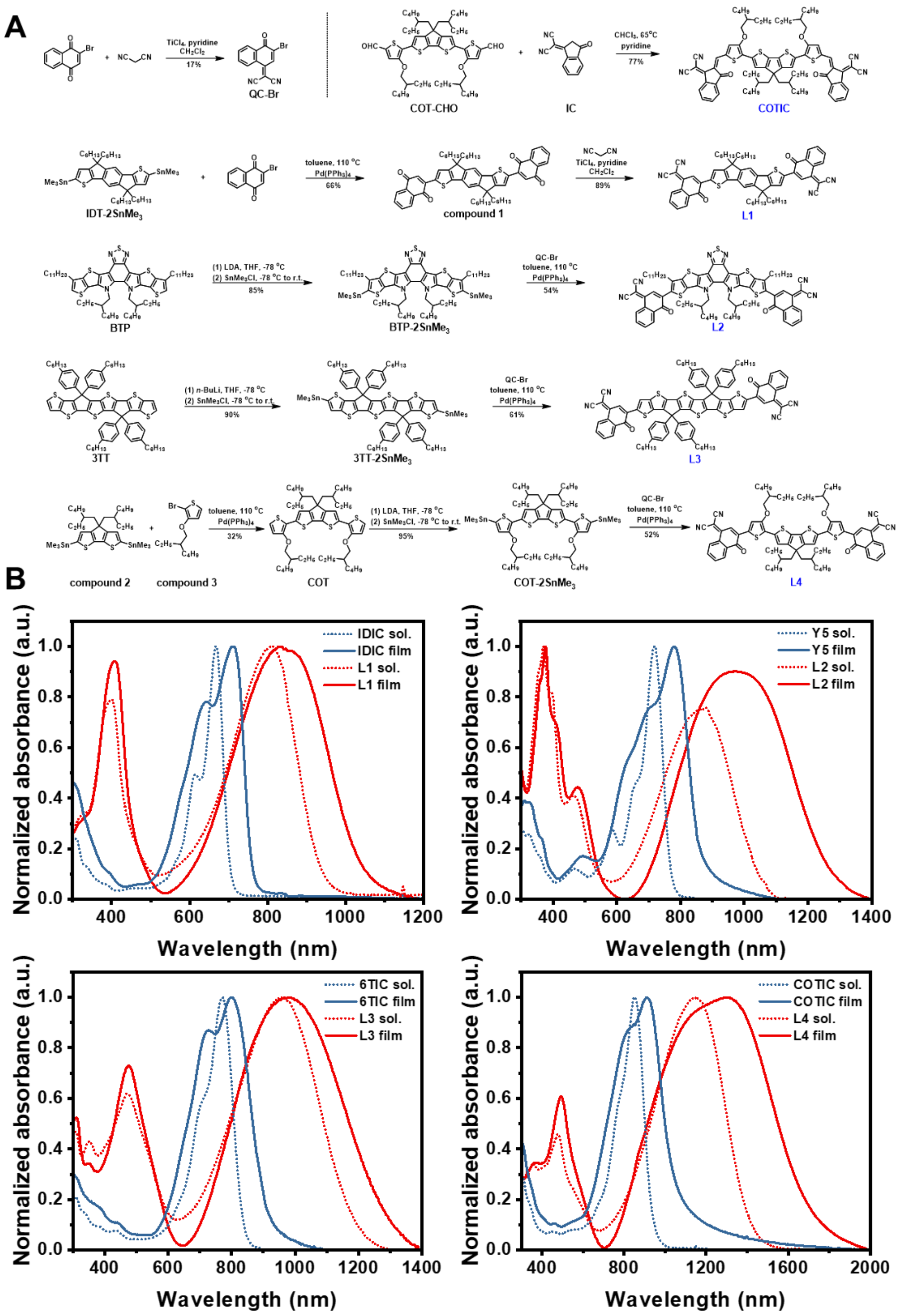

Fig. S3. Synthetic routes and absorption spectra. (A) Synthetic routes for QC-Br, COTIC, and L1-L4. **(B)** Absorption spectra of L1-L4 and their IC-based analogues (sol.: in dilute $CHCl_3$ solution; film: in $CHCl_3$-cast thin film).

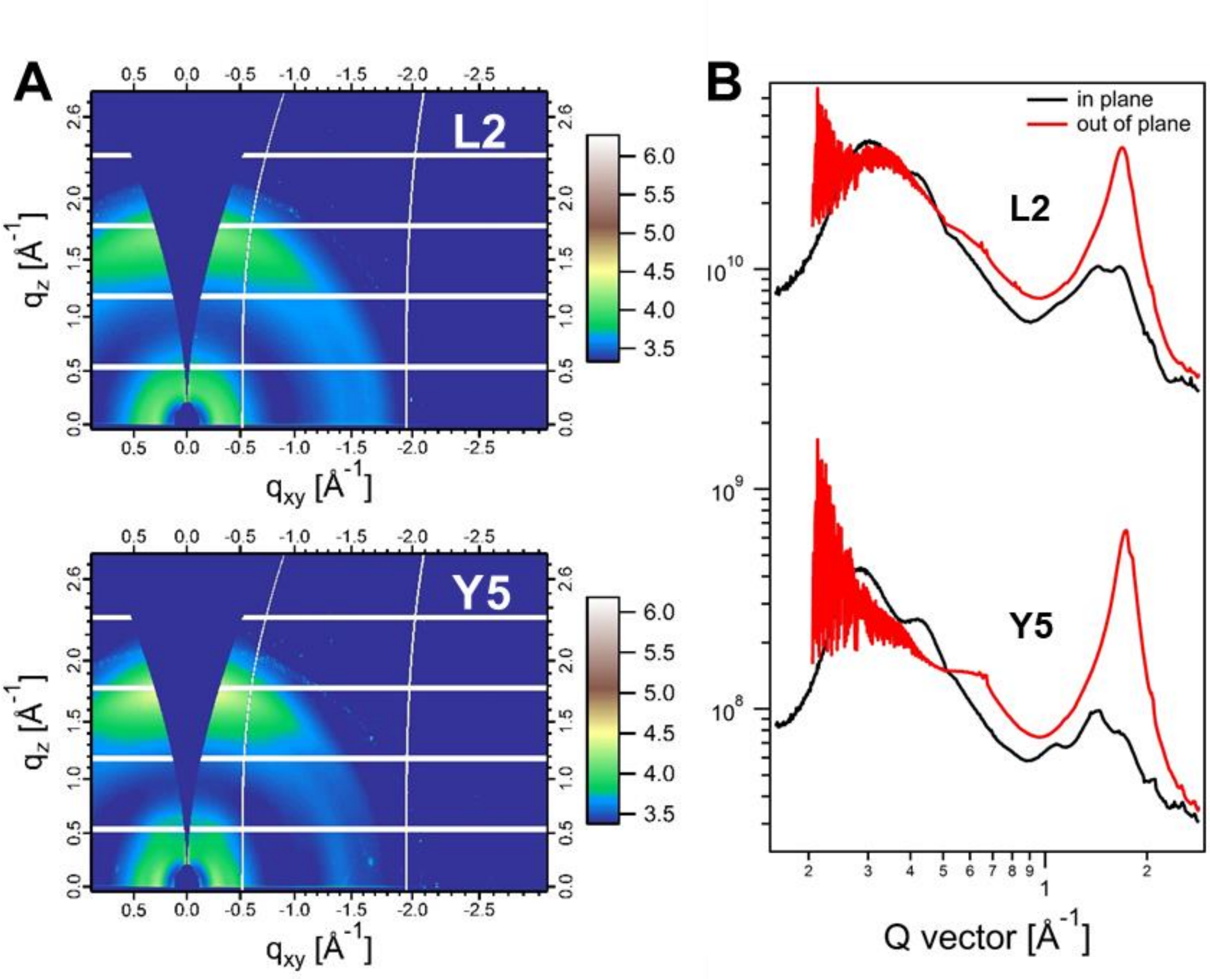

Fig. S4. **Morphology characterization of L2 and Y5 films.** (A) 2D GIWAXS patterns. (B) Intensity profiles in the in-plane and out-of-plane directions.

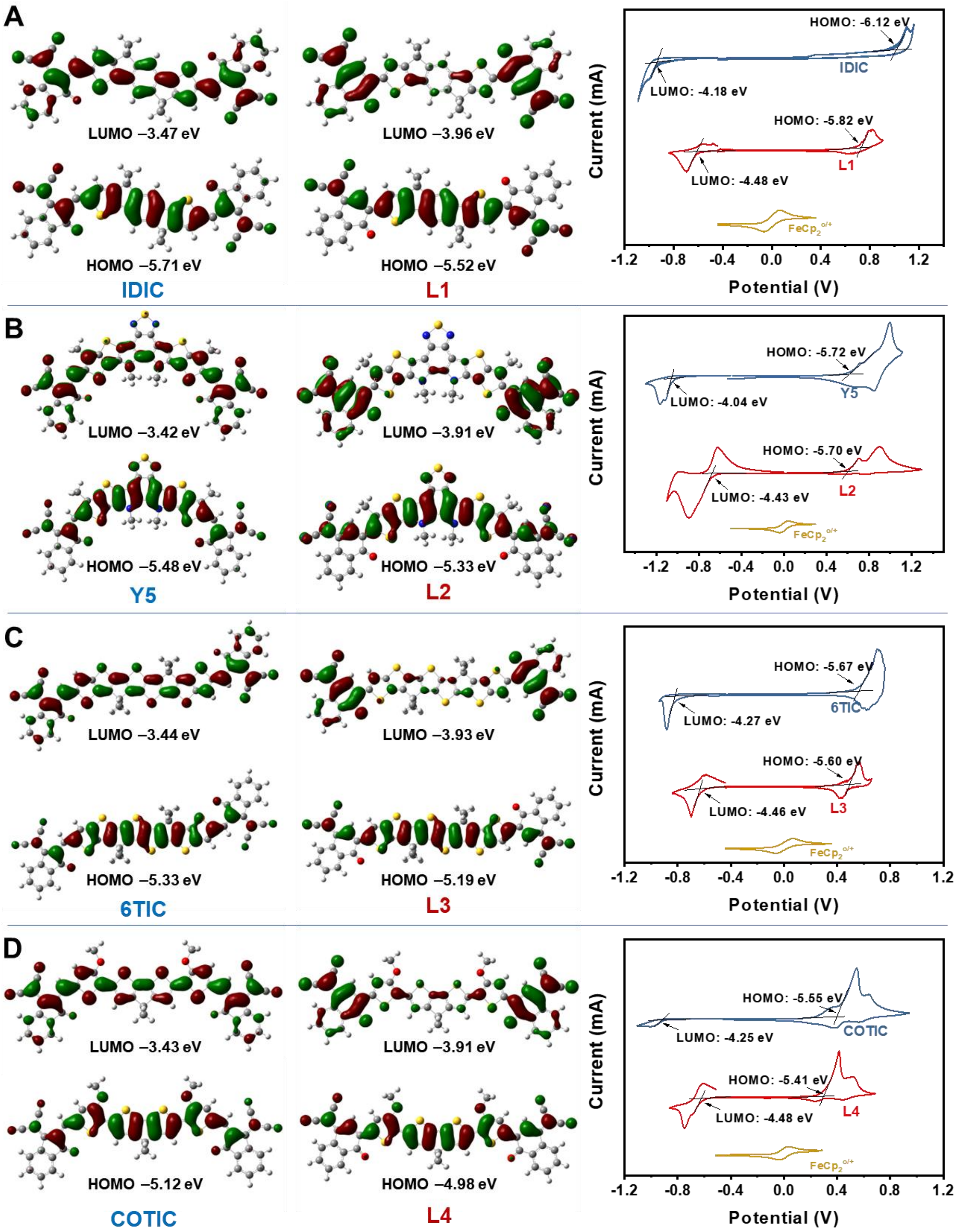

Fig. S5. Energy levels and electron distributions of frontier orbitals calculated by Gaussian 16 program at B3LYP/6-31G(d,p) level (all side chains are replaced by methyl groups) and cyclic voltammograms of (A) IDIC and L1, (B) Y5 and L2, (C) 6TIC and L3, and (D) COTIC and L4. Potential *vs.* $FeCp_2^{0/+}$

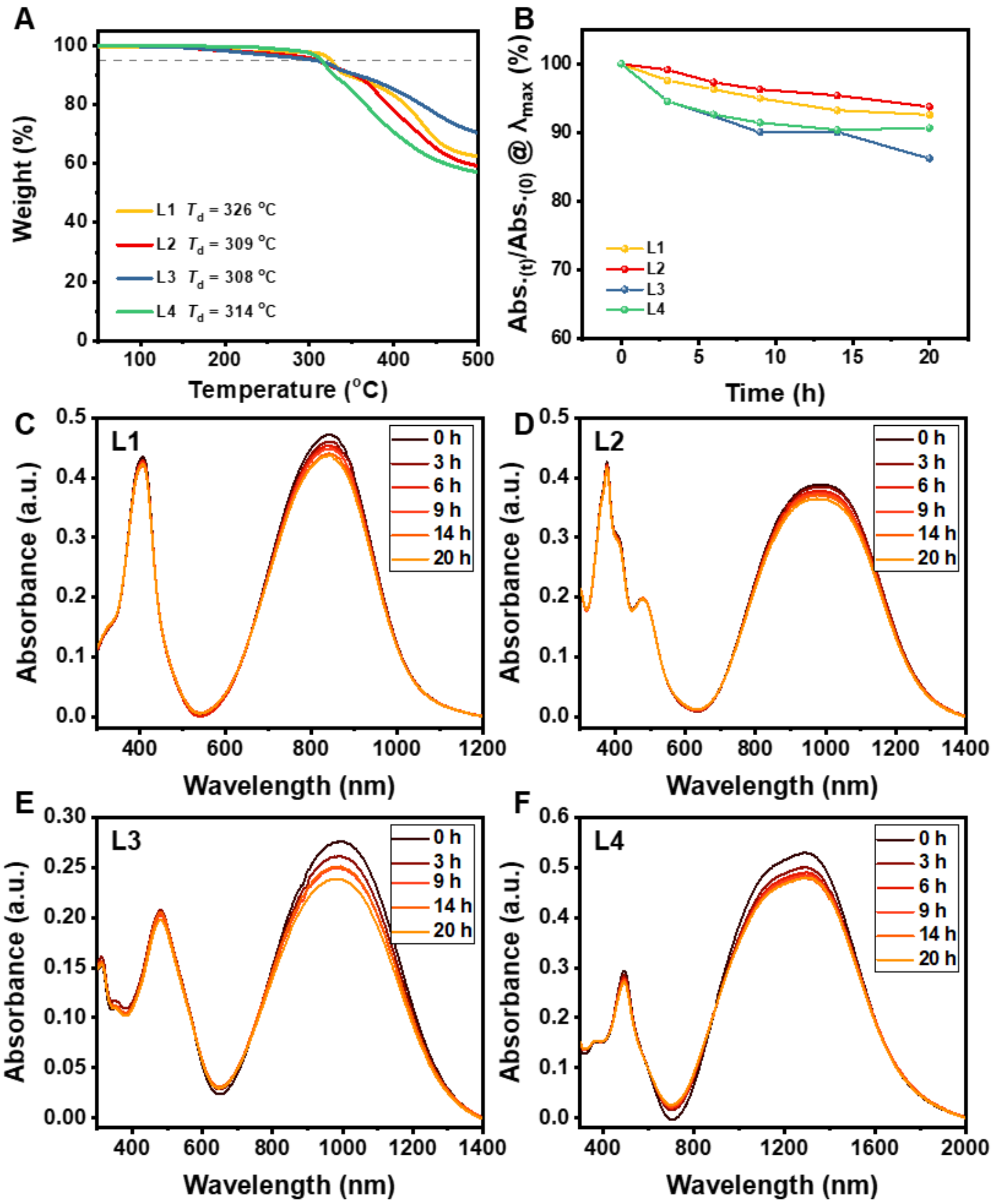

Fig. S6. The thermal and photo-oxidation stabilities of L1-L4. (A) The TGA curves of L1-L4. T_d is decomposition temperature at 5% weight loss. **(B)** The time-dependent absorption decays of L1-L4 films upon illumination with AM 1.5G (100 mW cm^{-2}) in the ambient air. λ_{max} refers to the wavelength of absorption peak in NIR region. The films were prepared by spin-coating L1-L4 solution (16 mg mL^{-1} in $CHCl_3$) at 2000 rpm for 30 s on the quartz substrate. **(C-F)** The variation of absorption spectra of L1-L4 films with the illumination time.

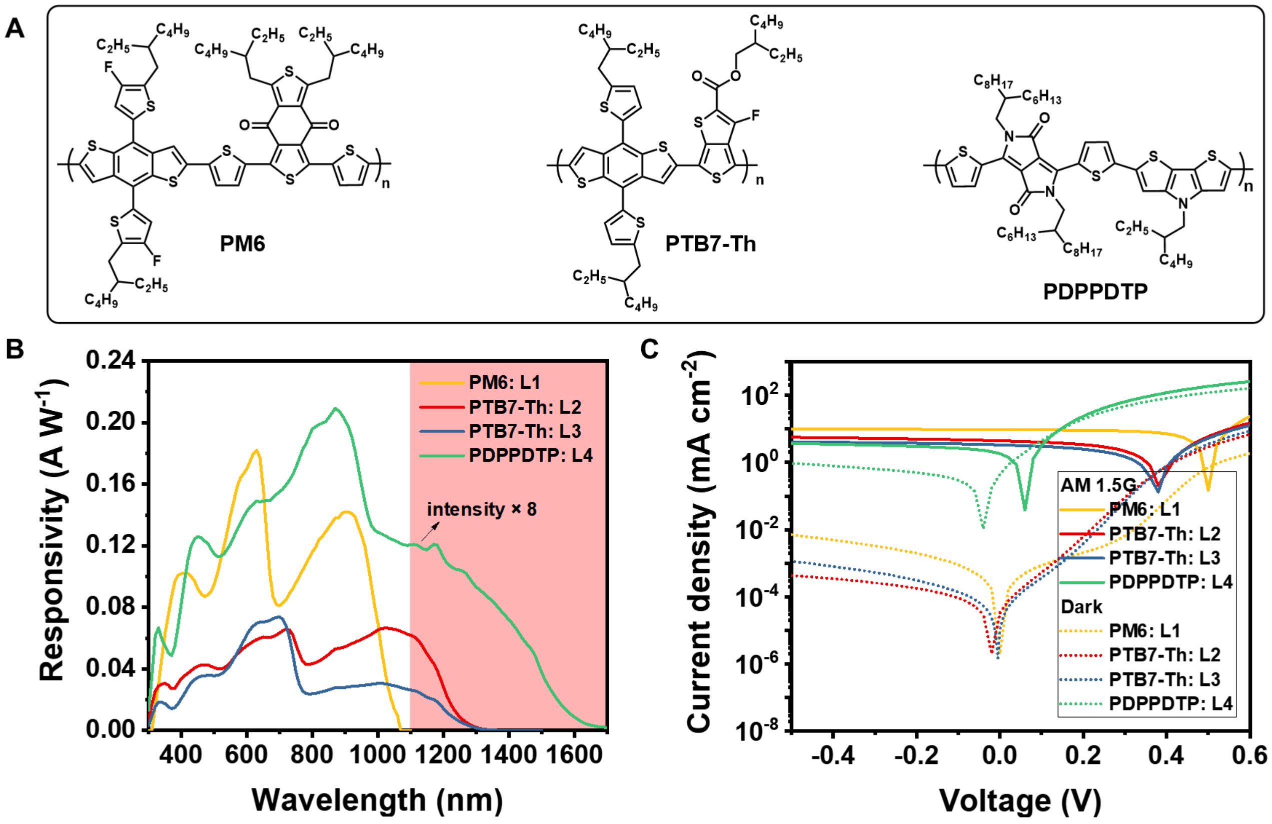

Fig. S7. Performance of NIR OPDs based on L1-L4 with device structure of ITO/PEDOT:PSS/active layer/DPO/Al. (A) Chemical structures of PM6, PTB7-Th, and PDPPDTP. **(B)** Responsivity under zero bias. **(C)** Characteristic *J*-*V* curves under AM 1.5G irradiation (100 mW cm^{-2}) and in the dark.

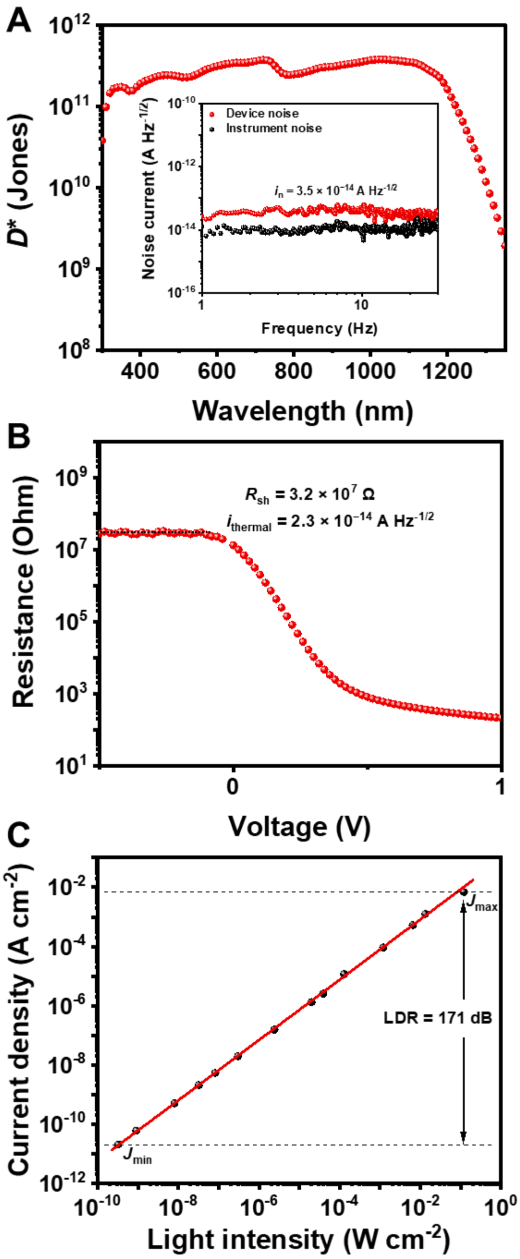

Fig. S8. Performance of NIR OPDs based on PTB7-Th: L2 with device structure of ITO/PEDOT:PSS/active layer/DPO/Al. (A) Specific detectivity under zero bias (inset: the measured device noise current under zero bias). **(B)** Differential resistance. Dashed line indicates the R_{sh} value. **(C)** The linear dynamic range measurement under the illumination at 1.0 μm of various light intensities under zero bias.

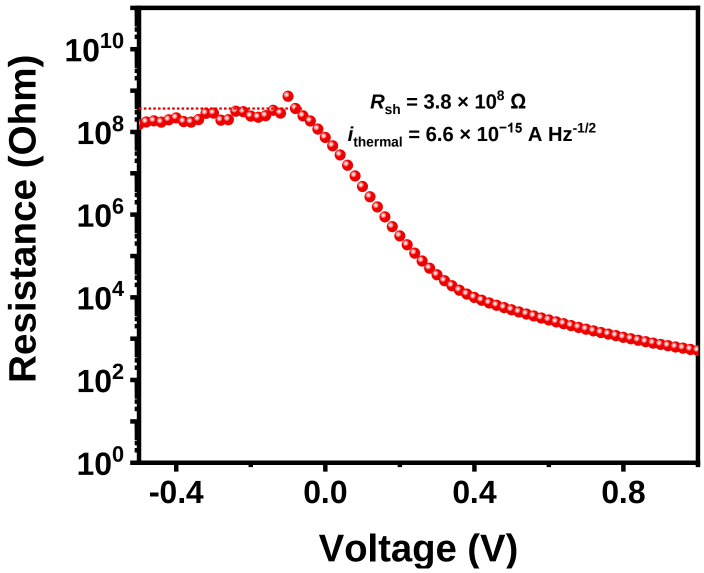

Fig. S9. Differential resistance of the NIR OPDs based on PTB7-Th: L2 with device structure of ITO/ZnO/active layer/MoO_3/Ag. Dashed line indicates the R_{sh} value.

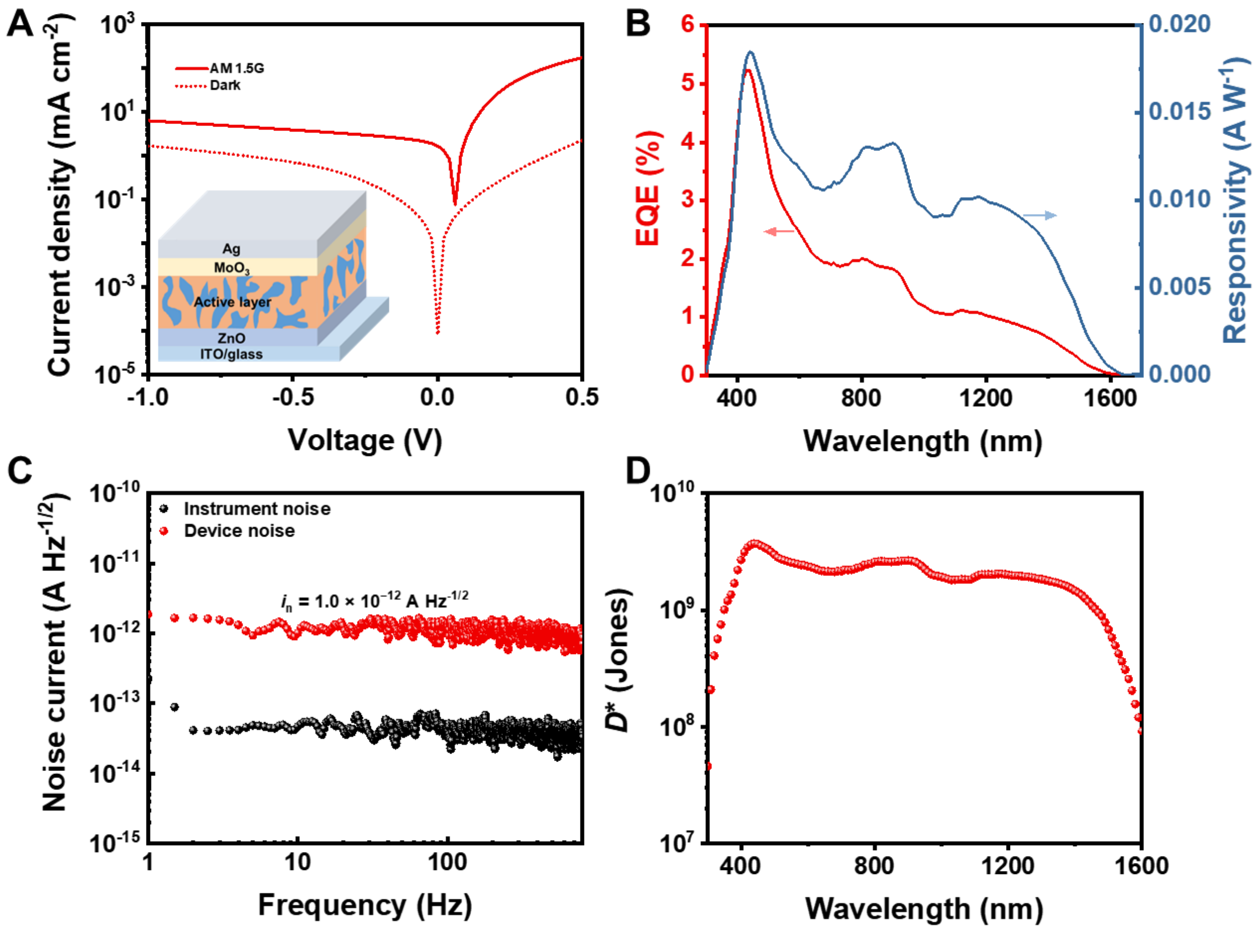

Fig. S10. Performance of NIR OPDs based on PDPPDTP: L4 with device structure of ITO/ZnO/active layer/MoO_3/Ag. (A) Characteristic *J*-*V* curves under AM 1.5G irradiation (100 mW cm^{-2}) and in the dark (inset: schematic diagram of the device structure). **(B)** EQE spectra and responsivity under zero bias. **(C)** The measured device noise current under zero bias. **(D)** Specific detectivity under zero bias.

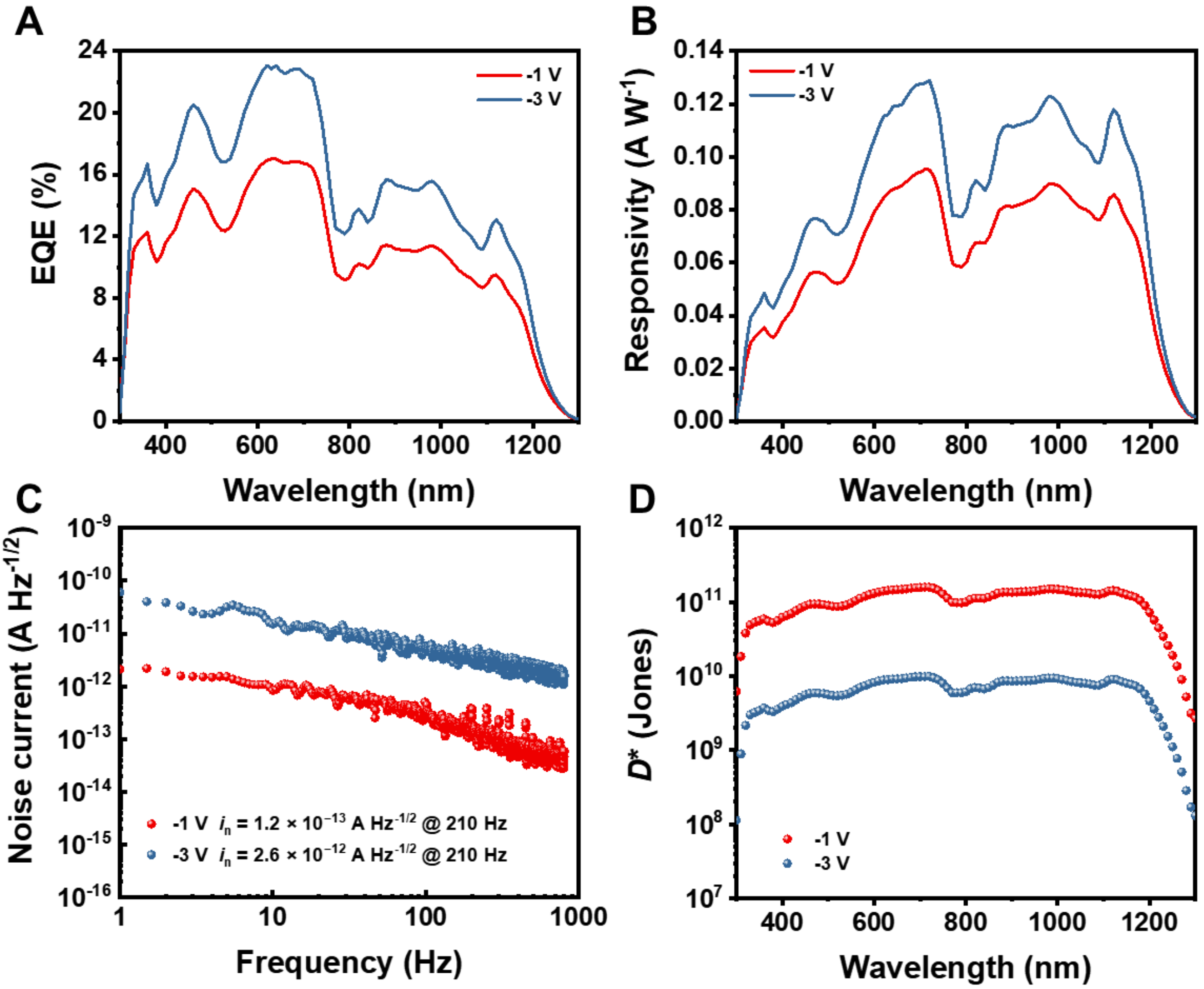

Fig. S11. Performance of NIR OPDs based on PTB7-Th: L2 with device structure of ITO/ZnO/active layer/MoO_3/Ag under different bias voltages. (A) EQE spectra. **(B)** Responsivity curves. **(C)** Noise currents. **(D)** Specific detectivity.

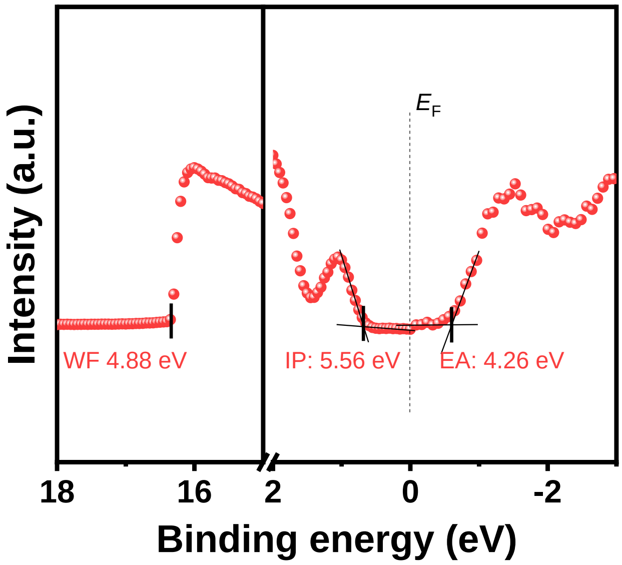

Fig. S12. UPS and LEIPS spectra of L2 with respect to the Fermi energy level (E_F) at 0 eV. IP refers to ionization potential and EA refers to electron affinity (work function (WF) values are labelled).

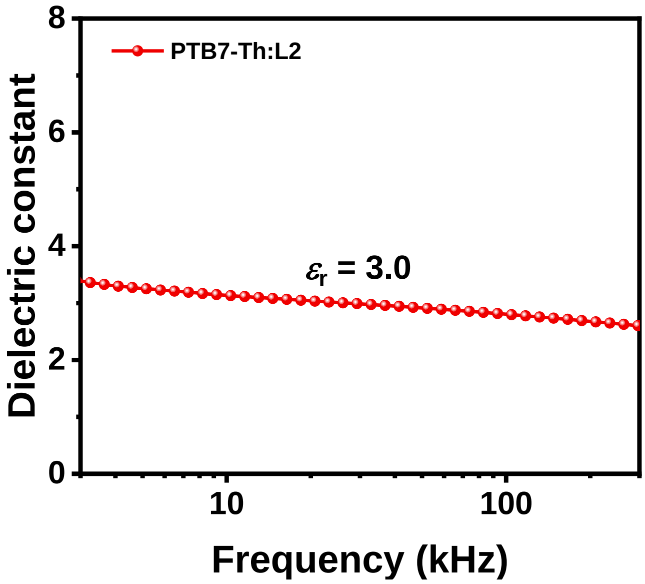

Fig. S13. Dielectric constant versus frequency of PTB7-Th: L2 blend film.

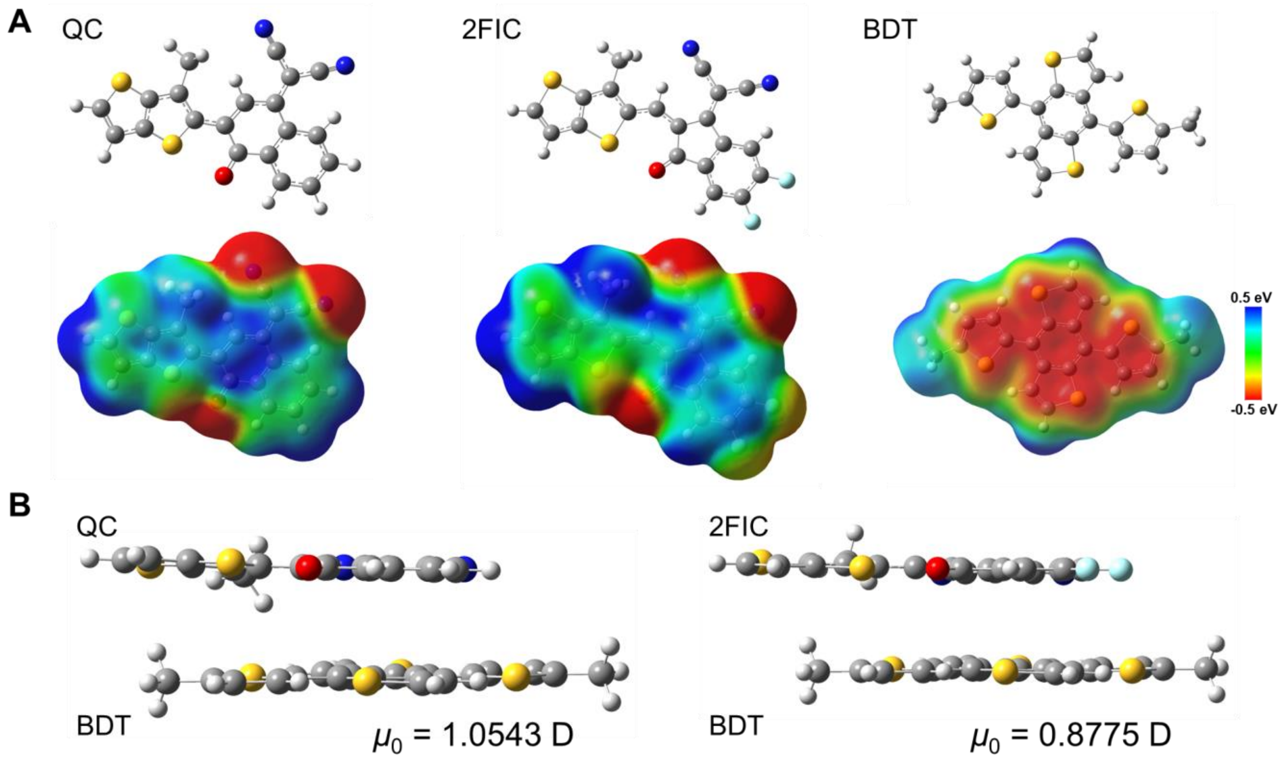

Fig. S14. **ESP induced dipole calculation. (A)** Rendering of the ESP on molecular surface for D unit of PTB7-Th (BDT), A units of L2 (QC) and Y6 (2FIC). **(B)** ESP induced dipole calculation for D-A units with face-on/face-on orientation in the designated y direction. The π-π stacking space of 3.5 Å is adopted. The dipole unit is shown in Debye (D). (Note: to get a better study of the impact of ESP on the intermolecular interactions, we make the molecular geometries very planar to avoid unexpected atomic interactions, and this approximation allows the field direction to be perpendicular to the plane) (*71*)

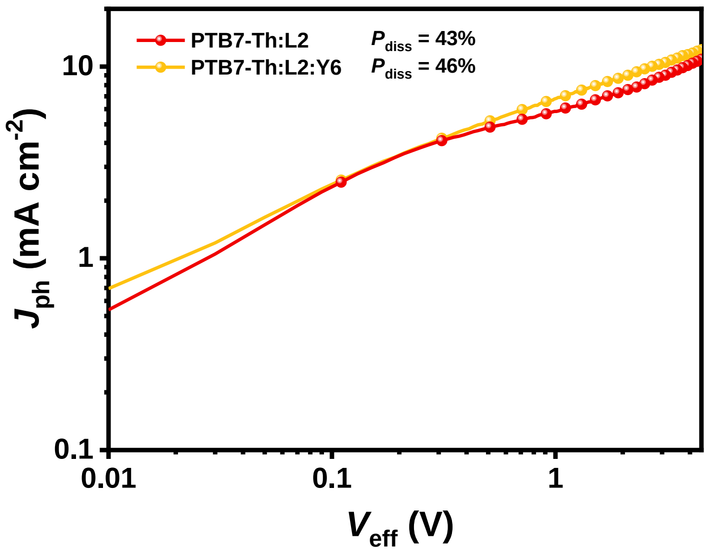

Fig. S15. J_{ph} versus V_{eff} characteristics of NIR OPDs based on PTB7-Th: L2 and PTBT-Th: L2: Y6 with device structure of ITO/ZnO/active layer/MoO_3/Ag. At a high effective voltage, it is assumed that all the photogenerated excitons are dissociated into free charge carriers and collected by electrodes, and the saturation photocurrent density (J_{sat}) is only limited by the total amount of absorbed incident photons, thus the ratio of J_{SC}/J_{sat} can be used to probe the exciton dissociation efficiency (P_{diss}) under short-circuit condition. (*72*) Here, considering that the J_{ph} values of the devices based on PTB7-Th:L2 and PTB7-Th:L2:Y6 did not reach saturation even at a high V_{eff} of 4 V, the pseudo J_{sat} values were obtained as 10.2 and 11.7 mA cm^{-2} at V_{eff} = 4 V for equitable comparison. (*73*) Correspondingly, the P_{diss} values were calculated as 43% for PTB7-Th:L2 based devices (J_{SC} = 4.4 mA cm^{-2}) and 46% for PTB7-Th:L2:Y6 based devices (J_{SC} = 5.4 mA cm^{-2}), respectively.

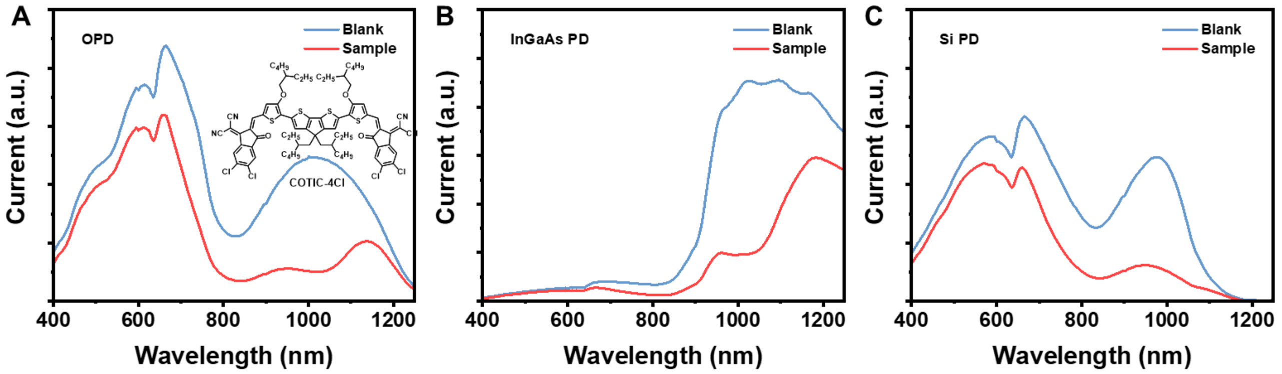

Fig. S16. The measured I_{sample} and I_{blank} of the spectrometer prototype using OPD (A), InGaAs PD (B), and Si PD (C) as the detector. The chemical structure of COTIC-4Cl is depicted as an inset in (A).

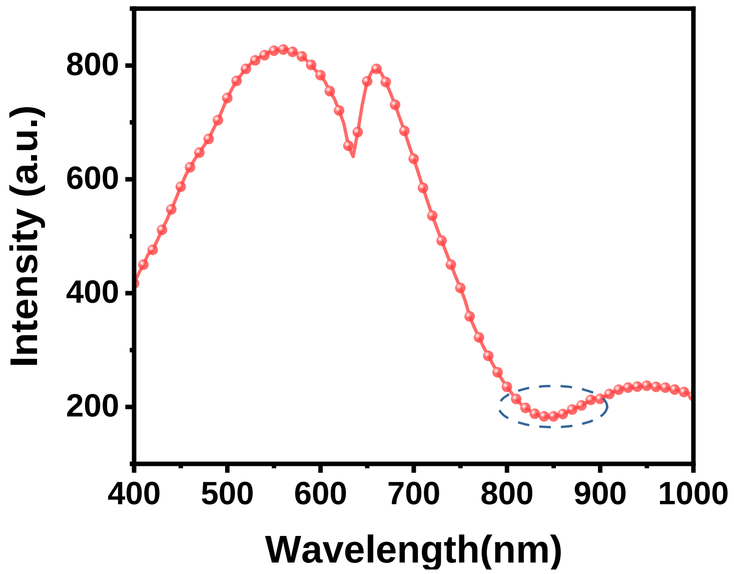

Fig. S17. The partial luminescence spectrum of tungsten lamp (CT-TH-150) used in spectrometer prototype.

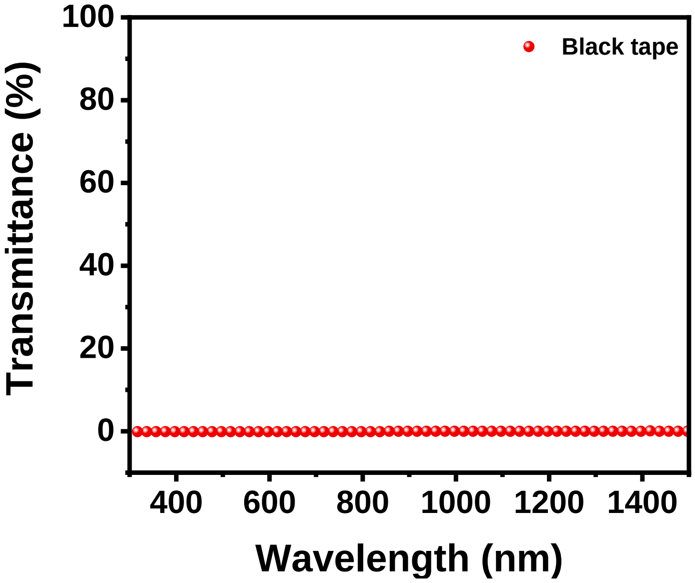

Fig. S18. The transmittance of the black tape used in the application of NIR imaging.

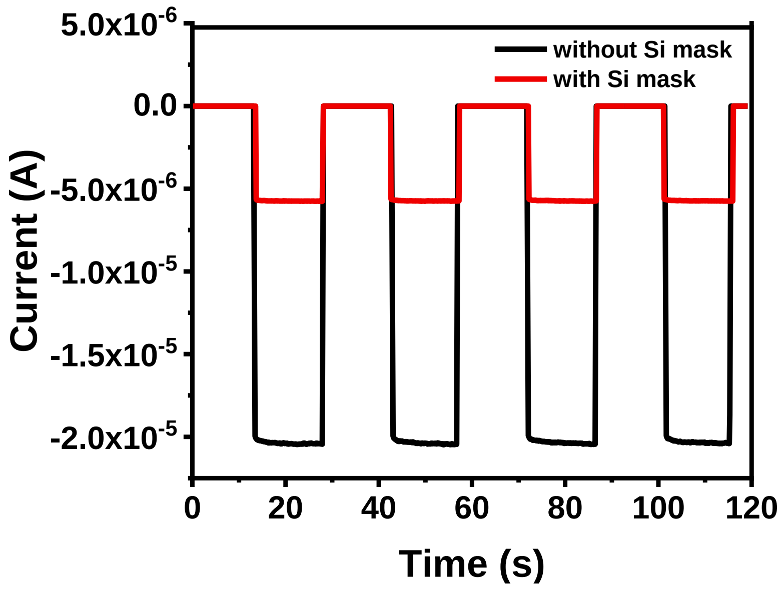

Fig. S19. The currents of the device with and w/o the Si mask under illumination (1.2 μm) at 21 mW cm^{-2}. The device is NIR OPDs based on PTB7-Th: L2 with device structure of ITO/PEDOT:PSS/active layer/DPO/Al.

Table S1. The TDOS and PDOS at LUMO energy level of the calculated IDT-based A-D-A molecules with varied "A" groups.

"A" group	TDOS	PDOS of IDT group	PDOS of "A" group	Contribution of "A" group
RCN	0.12131	0.04957	0.07174*	59%
IC	0.16852	0.04496	0.12356*	73%
2FIC	0.17324	0.04471	0.12853*	74%
2ClIC	0.17554	0.04425	0.13129*	75%
QC	0.13667	0.02335	0.11332	83%

*The PDOS data of these "A" groups all refer to the PDOS data of their vinyl-contained derivatives

Table S2. The summary of optical bandgap values of L1-L4 and their reference molecules which consist of the same "D" unit and varied "A" groups.

Compound	"D" unit	"A" group	E_g^{opt} (eV)*	Data source
IDIC	IDT	IC	1.65	**This work**
IDIC-4F	IDT	2FIC	1.62	*Chem. Commun.* 57, 5135-5138 (2021).
L1	IDT	QC	1.25	**This work**
Y5	BTP	IC	1.47	**This work**
Y6	BTP	2FIC	1.40	*Joule* 3, 1140-1151 (2019).
BTP-4Cl	BTP	2ClIC	1.38	*Nat. Commun.* 10, 2515 (2019).
L2	BTP	QC	1.03	**This work**
6TIC	3TT	IC	1.40	**This work**
F8IC	3TT	2FIC	1.33	*Adv. Mater.* 30, 1706571 (2018).
IXIC-4Cl	3TT	2ClIC	1.30	*Adv. Energy Mater.* 8, 1801203 (2018).
L3	3TT	QC	1.00	**This work**
COTIC	COT	IC	1.22	**This work**
COTIC-4F	COT	2FIC	1.10	*Adv. Energy Mater.* 8, 1801212 (2018).
COTIC-4Cl	COT	2ClIC	1.05	*Sci. China Mater.* 64, 2430-2438 (2021).
L4	COT	QC	0.77	**This work**

*The optical bandgap values were obtained from the absorption spectra of thin film in the previous literatures or in this work using the same method as shown in Fig. 2B.

Table S3. The summary of typical organic small-molecule semiconductors reported with small optical bandgap.

**Compound**	**E_g^{opt} (eV)**	**Type**	**Data source**
PDTTIC-4F	1.2	A-D-A molecule	*J. Am. Chem. Soc.* 143, 4281-4289 (2021).
COTIC-4F	1.1	A-D-A molecule	*Adv. Energy Mater.* 8, 1801212 (2018).
SSTI	0.98	A-D-A molecule	*Sol. RRL* 4, 2000286 (2020).
Pfused	0.8	Dye	*Adv. Mater.* 22, 2780-2783 (2010).
$OSnNcCl_2$	1.1	Dye	*Appl. Phys. Lett.* 95, 263302 (2009).
SQ-H	1.1	Dye	*Adv. Mater.* 33, 2100582 (2021).
BODIPY−BF2	1.2	Dye	*Adv. Electron. Mater.* 3, 1600430 (2017).
2-BF_4	0.8	Organic salts	*Adv. Optical Mater.* 4, 1028-1033 (2016).
Compound 2	0.99	Imide-based molecule	*Chem. Sci.* 7, 499-504 (2016).
FDT	1	Radical molecule	*Adv. Mater.* 27, 1718-1723 (2015).
L1	1.25	A-D-A molecule	**This work**
L2	1.03	A-D-A molecule	**This work**
L3	1.00	A-D-A molecule	**This work**
L4	0.77	A-D-A molecule	**This work**

Table S4. Figures of merit of reported NIR OPDs with photoresponse beyond 1.1 µm.

Photoactive material	Spectral range (nm)	λ* (nm)	Bias† (V)	Detectivity‡ (Jones)	Data source
CPDT-TQ: $PC_{71}BM$	600-1600	1180	0	5×10^{10}	*Adv. Funct. Mater.* 28, 1800391 (2018).
P1:$PC_{71}BM$	300-1540	1100	0	1.2×10^{11}	*Adv. Funct. Mater.* 28, 1805738 (2018).
PTTBAI:$PC_{71}BM$	400-1300	1000	0	$\sim 2 \times 10^{11}$	*Adv. Mater.* 32, 2003818 (2020).
PBTQ(OD):$PC_{71}BM$	400-1200	1000	0	$\sim 1 \times 10^{11}$	*Adv. Mater.* 32, 2003818 (2020).
PTTQ(HD):$PC_{71}BM$	400-1400	1200	0	$\sim 2 \times 10^{10}$	*Adv. Mater.* 32, 2003818 (2020).
PTTQn(HD):$PC_{71}BM$	400-1600	900	0	$\sim 4 \times 10^{7}$	*Adv. Mater.* 32, 2003818 (2020).
Pfused/C_{60}	400-1600	1350	0	(2.3×10^{10})	*Adv. Mater.* 22, 2780-2783 (2010).
Psub/C_{60}	400-1200	1090	0	(1.6×10^{11})	*Adv. Mater.* 22, 2780-2783 (2010).
Psub:PCBM:Bipy/C_{60}	400-1200	1150	0	(8.8×10^{11})	*Org. Electron.* 12, 869-873 (2011).
Pfused:PCBM:Bipy/C_{60}	400-1600	1400	0	(8.2×10^{10})	*Org. Electron.* 12, 869-873 (2011).
PDDTT:$PC_{61}BM$	400-1450	1400	0	(8×10^{9})	*J. Phys. Chem. C* 116, 13650-13653 (2012).
CPDT-*alt*-BSe:$PC_{71}BM$	600-1200	1000	0	(10^{12})	*ACS Appl. Mater. Interfaces* 9, 1654-1660 (2017).
FDT:$PC_{61}BM$	300-1500	1200	0	$(>10^{11})$	*Adv. Mater.* 27, 1718-1723 (2015).
1-TPFB/C_{60}	400-1460	1140	0	(5.3×10^{10})	*Adv. Optical Mater.* 4, 1028-1033 (2016).
P3:$PC_{71}BM$	600-1300	1330	0	(2×10^{11})	*Polym. Chem.* 8, 2922-2930 (2017).
P4:$PC_{71}BM$	600-1500	1200	0	(3×10^{11})	*Polym. Chem.* 8, 2922-2930 (2017).
PCE10: COTIC-4Cl:$PC_{71}BM$	300-1200	1100	–0.1	5.0×10^{12}	*Sci. China Mater.* 64, 2430-2438 (2021).
PTTBAI:$PC_{71}BM$	400-1300	1200	–2	$\sim 1 \times 10^{10}$	*Adv. Mater.* 32, 2003818 (2020).

PBTQ(OD):$PC_{71}BM$	400-1200	1000	–2	~9 × 10^{11}	*Adv. Mater.* 32, 2003818 (2020).
PTTQ(HD):$PC_{71}BM$	400-1400	1000	–2	~2 × 10^{11}	*Adv. Mater.* 32, 2003818 (2020).
PTTQn(HD):$PC_{71}BM$	400-1600	1000	–2	~8 × 10^{5}	*Adv. Mater.* 32, 2003818 (2020).
PDDTT:$PC_{61}BM$	300-1450	800	–0.1	(2.3 × 10^{13})	*Science* 325, 1665−1667 (2009).
PDPP-TIIG(P4):$PC_{61}BM$	300-1200	800	–0.1	(4.1 × 10^{11})	*Macromolecules* 48, 3941-3948 (2015).
P1:$PC_{61}BM$	300-1540	1150	–0.1	(2.2 × 10^{12})	*Adv. Optical Mater.* 6, 1800038 (2018).
P3:$PC_{61}BM$	300-1580	1040	–0.1	(9.1 × 10^{11})	*Adv. Optical Mater.* 6, 1800038 (2018)
P4:PCBM	300-1200	800	–0.1	(1.4 × 10^{12})	*Adv. Funct. Mater.* 24, 7605-7612 (2014).
PDT:$PC_{61}BM$	300-1600	900	–1	(1.9 × 10^{12})	*J. Mater. Chem. C* 5, 159-165 (2017).
PBBTPD:Tri-$PC_{61}BM$	350-2500	1500	–1	(2.2 × 10^{11})	*J. Mater. Chem. C* 6, 3634-3641 (2018).
PTB7-Th:L2	300-1300	1020	0	2.1 × 10^{12}	**This work**
PTB7-Th:L2:Y6	300-1300	1020	0	2.9 × 10^{12}	**This work**

*The corresponding wavelength of the maximum D^* in the NIR region. †The bias voltage applied on the devices. ‡The D^* values without bracket are calculated from the real-measured noise current and the D^* values in brackets are inferred from the white noise, leading to a possible overestimation of D^*.

REFERENCES AND NOTES

1. F. P. García de Arquer, A. Armin, P. Meredith, E. H. Sargent, Solution-processed semiconductors for next-generation photodetectors. *Nat. Rev. Mater.* **2**, 16100 (2017).

2. R. Saran, R. J. Curry, Lead sulphide nanocrystal photodetector technologies. *Nat. Photon.* **10**, 81–92 (2016).

3. F. H. Koppens, T. Mueller, P. Avouris, A. C. Ferrari, M. S. Vitiello, M. Polini, Photodetectors based on graphene, other two-dimensional materials and hybrid systems. *Nat. Nanotechnol.* **9**, 780–793 (2014).

4. Z. Wu, Y. Zhai, H. Kim, J. D. Azoulay, T. N. Ng, Emerging design and characterization guidelines for polymer-based infrared photodetectors. *Acc. Chem. Res.* **51**, 3144–3153 (2018).

5. J. Liu, F. Xia, D. Xiao, F. J. Garcia de Abajo, D. Sun, Semimetals for high-performance photodetection. *Nat. Mater.* **19**, 830–837 (2020).

6. C. Liu, J. Guo, L. Yu, J. Li, M. Zhang, H. Li, Y. Shi, D. Dai, Silicon/2D-material photodetectors: From near-infrared to mid-infrared. *Light. Sci. Appl.* **10**, 123 (2021).

7. J. Michel, J. Liu, L. C. Kimerling, High-performance Ge-on-Si photodetectors. *Nat. Photon.* **4**, 527–534 (2010).

8. A. Rogalski, Infrared detectors: Status and trends. *Prog. Quantum. Electron.* **27**, 59–210 (2003).

9. W. Pan, H. Wu, J. Luo, Z. Deng, C. Ge, C. Chen, X. Jiang, W.-J. Yin, G. Niu, L. Zhu, L. Yin, Y. Zhou, Q. Xie, X. Ke, M. Sui, J. Tang, $Cs_2AgBiBr_6$ single-crystal X-ray detectors with a low detection limit. *Nat. Photon.* **11**, 726–732 (2017).

10. J. Jiang, M. Xiong, K. Fan, C. Bao, D. Xin, Z. Pan, L. Fei, H. Huang, L. Zhou, K. Yao, X. Zheng, L. Shen, F. Gao, Synergistic strain engineering of perovskite single crystals for highly stable and sensitive X-ray detectors with low-bias imaging and monitoring. *Nat. Photon.* **16**, 575–581 (2022).

11. B. Xie, Z. Chen, L. Ying, F. Huang, Y. Cao, Near-infrared organic photoelectric materials for light-harvesting systems: Organic photovoltaics and organic photodiodes. *InfoMat.* **2**, 57–91 (2020).

12. Q. Li, Y. Guo, Y. Liu, Exploration of near-infrared organic photodetectors. *Chem. Mater.* **31**, 6359–6379 (2019).

13. P. Cheng, Y. Yang, Narrowing the band gap: The key to high-performance organic photovoltaics. *Acc. Chem. Res.* **53**, 1218–1228 (2020).

14. G. Li, W.-H. Chang, Y. Yang, Low-bandgap conjugated polymers enabling solution-processable tandem solar cells. *Nat. Rev. Mater.* **2**, 17043 (2017).

15. G. Qian, Z. Y. Wang, Near-infrared organic compounds and emerging applications. *Chem. Asian J.* **5**, 1006–1029 (2010).

16. L. Zheng, T. Zhu, W. Xu, L. Liu, J. Zheng, X. Gong, F. Wudl, Solution-processed broadband polymer photodetectors with a spectral response of up to 2.5 μm by a low bandgap donor–acceptor conjugated copolymer. *J. Mater. Chem. C* **6**, 3634–3641 (2018).

17. C. Yan, S. Barlow, Z. Wang, H. Yan, A. K. Y. Jen, S. R. Marder, X. Zhan, Non-fullerene acceptors for organic solar cells. *Nat. Rev. Mater.* **3**, 18003 (2018).

18. Y. J. Cheng, S. H. Yang, C. S. Hsu, Synthesis of conjugated polymers for organic solar cell applications. *Chem. Rev.* **109**, 5868–5923 (2009).

19. Y. Wu, H. T. Bai, Z. Y. Wang, P. Cheng, S. Y. Zhu, Y. F. Wang, W. Ma, X. W. Zhan, A planar electron acceptor for efficient polymer solar cells. *Energ. Environ. Sci.* **8**, 3215–3221 (2015).

20. Y. Lin, J. Wang, Z. G. Zhang, H. Bai, Y. Li, D. Zhu, X. Zhan, An electron acceptor challenging fullerenes for efficient polymer solar cells. *Adv. Mater.* **27**, 1170–1174 (2015).

21. J. Yuan, Y. Zhang, L. Zhou, G. Zhang, H.-L. Yip, T.-K. Lau, X. Lu, C. Zhu, H. Peng, P. A. Johnson, M. Leclerc, Y. Cao, J. Ulanski, Y. Li, Y. Zou, Single-junction organic solar cell with

over 15% efficiency using fused-ring acceptor with electron-deficient core. *Joule* **3**, 1140–1151 (2019).

22. J. Wang, X. Zhan, Fused-ring electron acceptors for photovoltaics and beyond. *Acc. Chem. Res.* **54**, 132–143 (2021).

23. K. Yamamoto, S. M. Quintero, S. Jinnai, E. Jeong, K. Matsuo, M. Suzuki, H. Yamada, J. Casado, Y. Ie, Cross-conjugated isothianaphthene quinoids: A versatile strategy for controlling electronic structures. *J. Mater. Chem. C* **10**, 4424–4433 (2022).

24. D. Meng, R. Zheng, Y. Zhao, E. Zhang, L. Dou, Y. Yang, Near-infrared materials: The turning point of organic photovoltaics. *Adv. Mater.* **34**, 2107330 (2022).

25. J. Lee, S. J. Ko, M. Seifrid, H. Lee, B. R. Luginbuhl, A. Karki, M. Ford, K. Rosenthal, K. Cho, T. Q. Nguyen, G. C. Bazan, Bandgap narrowing in non-fullerene acceptors: Single atom substitution leads to high optoelectronic response beyond 1000 nm. *Adv. Energy Mater.* **8**, 1801212 (2018).

26. Z. Wu, Y. Zhai, W. Yao, N. Eedugurala, S. Zhang, L. Huang, X. Gu, J. D. Azoulay, T. N. Ng, The role of dielectric screening in organic shortwave infrared photodiodes for spectroscopic image sensing. *Adv. Funct. Mater.* **28**, 1805738 (2018).

27. Z. Wu, W. Yao, A. E. London, J. D. Azoulay, T. N. Ng, Elucidating the detectivity limits in shortwave infrared organic photodiodes. *Adv. Funct. Mater.* **28**, 1800391 (2018).

28. S. Gielen, C. Kaiser, F. Verstraeten, J. Kublitski, J. Benduhn, D. Spoltore, P. Verstappen, W. Maes, P. Meredith, A. Armin, K. Vandewal, Intrinsic detectivity limits of organic near-infrared photodetectors. *Adv. Mater.* **32**, 2003818 (2020).

29. Judson, Datasheet of J16. www.teledynejudson.com/prods/Documents/Ge_shortform_August2004.pdf.

30. Judson, Datasheet of J23. www.teledynejudson.com/prods/Documents/InGaAs_shortform_DEC2004_rev2.pdf.

31. Thorlabs, Product page of FGA21.
www.thorlabschina.cn/thorProduct.cfm?partNumber=FGA21.

32. Hamamatsu, Datasheet of G8370-81/-82/-83/-85.
www.hamamatsu.com.cn/content/dam/hamamatsu-photonics/sites/documents/99_SALES_LIBRARY/ssd/g8370-81_etc_kird1064e.pdf.

33. L. R. Melby, R. J. Harder, W. R. Hertler, W. Mahler, R. E. Benson, W. E. Mochel, Substituted quinodimethans. II. Anion-radical derivatives and complexes of 7,7,8,8-tetracyanoquinodimethan. *J. Am. Chem. Soc.* **84**, 3374–3387 (1962).

34. Y. Sun, Y. Guo, Y. Liu, Design and synthesis of high performance π-conjugated materials through antiaromaticity and quinoid strategy for organic field-effect transistors. *Mater. Sci. Eng. R* **136**, 13–26 (2019).

35. J. López, F. de la Cruz, Y. Alcaraz, F. Delgado, M. A. Vázquez, Quinoid systems in chemistry and pharmacology. *Med. Chem. Res.* **24**, 3599–3620 (2015).

36. Y. Cui, P. Zhu, X. Shi, X. Liao, Y. Chen, Theoretical study of excited state charge transfer characteristics based on A–D–A and A–DA′D–A type nonfullerene acceptors. *J. Phys. Chem. C* **125**, 10250–10259 (2021).

37. K. Takimiya, K. Kawabata, Thienoquinoidal system: Promising molecular architecture for optoelectronic applications. *J. Synth. Org. Chem. Jpn.* **76**, 1176–1184 (2018).

38. Y. Lin, Q. He, F. Zhao, L. Huo, J. Mai, X. Lu, C. J. Su, T. Li, J. Wang, J. Zhu, Y. Sun, C. Wang, X. Zhan, A facile planar fused-ring electron acceptor for As-Cast polymer solar cells with 8.71% efficiency. *J. Am. Chem. Soc.* **138**, 2973–2976 (2016).

39. J. Yuan, Y. Zhang, L. Zhou, C. Zhang, T. K. Lau, G. Zhang, X. Lu, H. L. Yip, S. K. So, S. Beaupre, M. Mainville, P. A. Johnson, M. Leclerc, H. Chen, H. Peng, Y. Li, Y. Zou, Fused benzothiadiazole: A building block for n-type organic acceptor to achieve high-performance organic solar cells. *Adv. Mater.* **31**, 1807577 (2019).

40. X. L. Shi, J. D. Chen, K. Gao, L. J. Zuo, Z. Y. Yao, F. Liu, J. X. Tang, A. K. Y. Jen, Terthieno[3,2-*b*]thiophene (6T) based low bandgap fused-ring electron acceptor for highly efficient solar cells with a high short-circuit current density and low open-circuit voltage loss. *Adv. Energy Mater.* **8**, 1702831 (2018).

41. J. H. Kim, T. Schembri, D. Bialas, M. Stolte, F. Wurthner, Slip-stacked J-aggregate materials for organic solar cells and photodetectors. *Adv. Mater.* **34**, 2104678 (2022).

42. A. Hexemer, W. Bras, J. Glossinger, E. Schaible, E. Gann, R. Kirian, A. MacDowell, M. Church, B. Rude, H. Padmore, A SAXS/WAXS/GISAXS beamline with multilayer monochromator. *J. Phys. Conf. Ser.* **247**, 012007 (2010).

43. N. J. Hestand, F. C. Spano, Molecular aggregate photophysics beyond the Kasha model: Novel design principles for organic materials. *Acc. Chem. Res.* **50**, 341–350 (2017).

44. G. Yu, J. Gao, J. C. Hummelen, F. Wudl, A. J. Heeger, Polymer photovoltaic cells—Enhanced efficiencies via a network of internal donor-acceptor heterojunctions. *Science* **270**, 1789–1791 (1995).

45. P. Peumans, S. Uchida, S. R. Forrest, Efficient bulk heterojunction photovoltaic cells using small-molecular-weight organic thin films. *Nature* **425**, 158–162 (2003).

46. W. Y. Tan, R. Wang, M. Li, G. Liu, P. Chen, X. C. Li, S. M. Lu, H. L. Zhu, Q. M. Peng, X. H. Zhu, W. Chen, W. C. H. Choy, F. Li, J. B. Peng, Y. Cao, Lending triarylphosphine oxide to phenanthroline: A facile approach to high-performance organic small-molecule cathode interfacial material for organic photovoltaics utilizing air-stable cathodes. *Adv. Funct. Mater.* **24**, 6540–6547 (2014).

47. K. H. Hendriks, W. Li, M. M. Wienk, R. A. Janssen, Small-bandgap semiconducting polymers with high near-infrared photoresponse. *J. Am. Chem. Soc.* **136**, 12130–12136 (2014).

48. S. H. Liao, H. J. Jhuo, Y. S. Cheng, S. A. Chen, Fullerene derivative-doped zinc oxide nanofilm as the cathode of inverted polymer solar cells with low-bandgap polymer (PTB7-Th) for high performance. *Adv. Mater.* **25**, 4766–4771 (2013).

49. Y. Fang, A. Armin, P. Meredith, J. Huang, Accurate characterization of next-generation thin-film photodetectors. *Nat. Photon.* **13**, 1–4 (2019).

50. R. D. Jansen-van Vuuren, A. Armin, A. K. Pandey, P. L. Burn, P. Meredith, Organic photodiodes: The future of full color detection and image sensing. *Adv. Mater.* **28**, 4766–4802 (2016).

51. F.-H. Canek, C. Wen-Fang, M. K. Talha, D. Larissa, L. Julia, A. L. Felipe, A. R.-T. Victor, K. Bernard, Large-area low-noise flexible organic photodiodes for detecting faint visible light. *Science* **370**, 698–701 (2020).

52. G. Liu, T. Li, X. Zhan, H. Wu, Y. Cao, High-sensitivity visible–near infrared organic photodetectors based on non-fullerene acceptors. *ACS Appl. Mater. Interfaces* **12**, 17769–17775 (2020).

53. Y. Fang, J. Huang, Resolving weak light of sub-picowatt per square centimeter by hybrid perovskite photodetectors enabled by noise reduction. *Adv. Mater.* **27**, 2804–2810 (2015).

54. C. Li, H. Wang, F. Wang, T. Li, M. Xu, H. Wang, Z. Wang, X. Zhan, W. Hu, L. Shen, Ultrafast and broadband photodetectors based on a perovskite/organic bulk heterojunction for large-dynamic-range imaging. *Light Sci. Appl.* **9**, 31 (2020).

55. Hamamatsu, Datasheet of Si photodiode S1787 series. www.hamamatsu.com.cn/content/dam/hamamatsu-photonics/sites/documents/99_SALES_LIBRARY/ssd/s1787_series_kspd1038e.pdf.

56. J. Barton, R. Cannata, S. Petronio, InGaAs NIR focal plane arrays for imaging and DWDM applications. *Proc. SPIE* **4721**, 37–47 (2002).

57. J. V. Caspar, T. J. Meyer, Application of the energy gap law to nonradiative, excited-state decay. *J. Phys. Chem.* **87**, 952–957 (1983).

58. E. Collado-Fregoso, S. N. Pugliese, M. Wojcik, J. Benduhn, E. Bar-Or, L. Perdigon Toro, U. Hormann, D. Spoltore, K. Vandewal, J. M. Hodgkiss, D. Neher, Energy-gap law for photocurrent generation in fullerene-based organic solar cells: The case of low-donor-content blends. *J. Am. Chem. Soc.* **141**, 2329–2341 (2019).

59. Z. Wu, N. Li, N. Eedugurala, J. D. Azoulay, D.-S. Leem, T. N. Ng, Noise and detectivity limits in organic shortwave infrared photodiodes with low disorder. *npj Flexible Electron.* **4**, 6 (2020).

60. S. Karuthedath, J. Gorenflot, Y. Firdaus, N. Chaturvedi, C. S. P. De Castro, G. T. Harrison, J. I. Khan, A. Markina, A. H. Balawi, T. A. D. Pena, W. Liu, R. Z. Liang, A. Sharma, S. H. K. Paleti, W. Zhang, Y. Lin, E. Alarousu, D. H. Anjum, P. M. Beaujuge, S. De Wolf, I. McCulloch, T. D. Anthopoulos, D. Baran, D. Andrienko, F. Laquai, Intrinsic efficiency limits in low-bandgap non-fullerene acceptor organic solar cells. *Nat. Mater.* **20**, 378–384 (2021).

61. X. Li, Q. Zhang, J. Yu, Y. Xu, R. Zhang, C. Wang, H. Zhang, S. Fabiano, X. Liu, J. Hou, F. Gao, M. Fahlman, Mapping the energy level alignment at donor/acceptor interfaces in non-fullerene organic solar cells. *Nat. Commun.* **13**, 2046 (2022).

62. T. Li, K. Wang, G. Cai, Y. Li, H. Liu, Y. Jia, Z. Zhang, X. Lu, Y. Yang, Y. Lin, Asymmetric glycolated substitution for enhanced permittivity and ecocompatibility of high-performance photovoltaic electron acceptor. *JACS Au* **1**, 1733–1742 (2021).

63. Y. Song, Z. Zhong, P. He, G. Yu, Q. Xue, L. Lan, F. Huang, Doping compensation enables high-detectivity infrared organic photodiodes for image sensing. *Adv. Mater.* **34**, 2201827 (2022).

64. Z. M. Zhong, F. Peng, L. Ying, G. Yu, F. Huang, Y. Cao, Ternary organic photodiodes with spectral response from 300 to 1200 nm for spectrometer application. *Sci. China Mater.* **64**, 2430–2438 (2021).

65. M. Helgesen, F. C. Krebs, Photovoltaic performance of polymers based on dithienylthienopyrazines bearing thermocleavable benzoate esters. *Macromolecules* **43**, 1253–1260 (2010).

66. H. Yao, Y. Chen, Y. Qin, R. Yu, Y. Cui, B. Yang, S. Li, K. Zhang, J. Hou, Design and synthesis of a low bandgap small molecule acceptor for efficient polymer solar cells. *Adv. Mater.* **28**, 8283–8287 (2016).

67. G. W. T. M. J. Frisch, H. B. Schlegel, G. E. Scuseria, M. A. Robb, J. R. Cheeseman, G. Scalmani, V. Barone, B. Mennucci, G. A. Petersson, H. Nakatsuji, M. Caricato, X. Li, H. P. Hratchian, A. F. Izmaylov, J. Bloino, G. Zheng, J. L. Sonnenberg, M. Hada, M. Ehara, K. Toyota, R. Fukuda, J. Hasegawa, M. Ishida, T. Nakajima, Y. Honda, O. Kitao, H. Nakai, T. Vreven, J. A. Montgomery Jr., J. E. Peralta, F. Ogliaro, M. J. Bearpark, J. Heyd, E. N. Brothers, K. N. Kudin, V. N. Staroverov, R. Kobayashi, J. Normand, K. Raghavachari, A. P. Rendell, J. C. Burant, S. S. Iyengar, J. Tomasi, M. Cossi, N. Rega, N. J. Millam, M. Klene, J. E. Knox, J. B. Cross, V. Bakken, C. Adamo, J. Jaramillo, R. Gomperts, R. E. Stratmann, O. Yazyev, A. J. Austin, R. Cammi, C. Pomelli, J. W. Ochterski, R. L. Martin, K. Morokuma, V. G. Zakrzewski, G. A. Voth, P. Salvador, J. J. Dannenberg, S. Dapprich, A. D. Daniels, Ö. Farkas, J. B. Foresman, J. V. Ortiz, J. Cioslowski and D. J. Fox, Gaussian 09, Revision A.01 ed., Gaussian, Inc., Wallingford CT, USA. (2009).

68. T. Lu, F. Chen, Multiwfn: A multifunctional wavefunction analyzer. *J. Comput. Chem.* **33**, 580–592 (2012).

69. A. Salleo, Electronic Traps in Organic Semiconductors, in *Organic Electronics: Emerging Concepts and Technologies,* F. Cicoira, C. Santato, Eds. (Wiley, 2013), chap. 14, pp. 341–380.

70. R. A. Street, Y. Yang, B. C. Thompson, I. McCulloch, Capacitance spectroscopy of light induced trap states in organic solar cells. *J. Phys. Chem. C* **120**, 22169–22178 (2016).

71. H. Yao, Y. Cui, D. Qian, C. S. Ponseca Jr., A. Honarfar, Y. Xu, J. Xin, Z. Chen, L. Hong, B. Gao, R. Yu, Y. Zu, W. Ma, P. Chabera, T. Pullerits, A. Yartsev, F. Gao, J. Hou, 14.7%

Efficiency organic photovoltaic cells enabled by active materials with a large electrostatic potential difference. *J. Am. Chem. Soc.* **141**, 7743–7750 (2019).

72. L. Lu, T. Xu, W. Chen, E. S. Landry, L. Yu, Ternary blend polymer solar cells with enhanced power conversion efficiency. *Nat. Photon.* **8**, 716–722 (2014).

73. J. Sun, X. Ma, Z. Zhang, J. Yu, J. Zhou, X. Yin, L. Yang, R. Geng, R. Zhu, F. Zhang, W. Tang, Dithieno[3,2-*b*:2',3'-*d*]pyrrol fused nonfullerene acceptors enabling over 13% efficiency for organic solar cells. *Adv. Mater.* **30**, e1707150 (2018).
